# Potassium Alloy Reference Electrodes for Potassium-Ion Batteries: The K-In and K-Bi Systems

**DOI:** 10.1021/acsmaterialslett.4c01219

**Published:** 2024-08-30

**Authors:** Ben Jagger, Jack Aspinall, Souhardh Kotakadi, John Cattermull, Shobhan Dhir, Mauro Pasta

**Affiliations:** † Department of Materials, University of Oxford, Oxford OX1 3PH, U.K.; ‡ Inorganic Chemistry Laboratory, Department of Chemistry, University of Oxford, Oxford OX1 3QR, U.K.

## Abstract

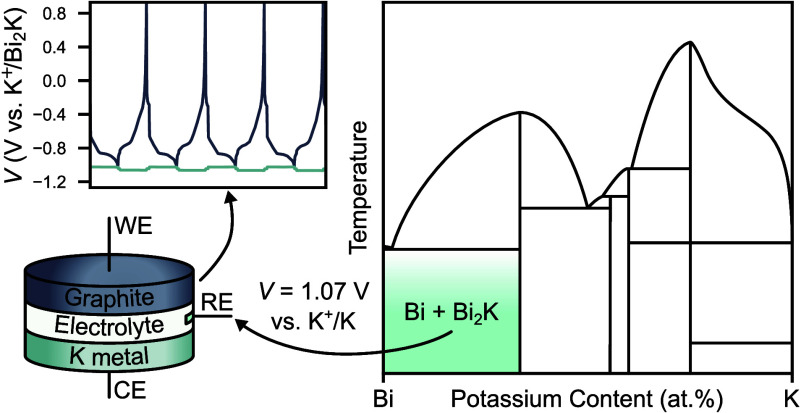

Potassium-ion batteries (KIBs) are a promising alternative to conventional lithium-ion batteries with reduced critical mineral dependency but accurate three-electrode characterization is hindered by the lack of a suitable reference electrode. Potassium metal is frequently used as a reference electrode out of necessity, but its high reactivity and unstable potential limit its reliability. Here we investigate the K-In and K-Bi alloy systems, synthesize two-phase In-In_4_K and Bi-Bi_2_K alloys, and identify Bi-Bi_2_K as a promising material owing to its stable potential of 1.07 V vs K^+^/K. We prove the use of Bi-Bi_2_K as a reference electrode by cycling graphite in three-electrode cells and demonstrate that it results in significantly less electrolyte reduction than potassium metal, facilitating the accurate electrochemical characterization necessary to accelerate KIB development.

Driven predominantly by widespread adoption of electric vehicles (EVs), global battery demand is projected to increase rapidly in the coming years, growing from 1 TWh yr^–1^ in 2023 to 13 TWh yr^–1^ by 2050 to meet net zero targets.^1^ With lithium-ion batteries (LIBs) predicted to make up over 90% of the market share, this will pose significant strain on the supply chains for critical minerals necessary for LIBs, including copper, lithium, nickel and cobalt. Expected copper and lithium supply, especially, is set to fall far short of demand,^1^ requiring the development of new battery technologies with reduced critical mineral dependencies, including sodium-ion batteries (NIBs) and potassium-ion batteries (KIBs).^2^

NIBs and KIBs are both attractive owing to the low cost and high abundance of sodium and potassium resources, and their inability to alloy with aluminum further enables the use of aluminum current collectors, rather than the copper required in LIBs.^3^ Additionally, K^+^ reversibly intercalates into graphite,^4^ while Na^+^ cannot, so one of the primary anode materials for KIBs is already produced at commercial scale, highlighting a key advantage of KIBs. The most promising cathode materials for KIBs also do not rely on critical minerals.^5,6^

KIBs are still in the early stages of development, requiring further electrolyte and electrode optimization before they can be commercialized.^7,8^ Accurate electrochemical characterization will be vital to guide such optimization, necessitating the use of three-electrode cells to accurately assign electrochemical behavior to each electrode. A key requirement for this is a suitable reference electrode, which should have a stable, well-defined potential, should be chemically inert, and should operate within the electrochemical stability window of KIB electrolytes.

There are few available reports on the production of reference electrodes for KIBs,^3,9^ so many studies use potassium metal, which does not satisfy the requirements of a reference electrode. The potential of potassium metal suffers from irreproducibility and it can drift over time,^10^ which may be caused by the presence of high concentrations of sodium and other impurities.^11^ Potassium metal pretreatment has therefore been necessary to achieve satisfactory reliability of rest potential.^10,11^ Potassium metal is also highly reactive, decomposing all known electrolytes on contact and forming resistive solid electrolyte interphases (SEIs). Electrolyte decomposition products formed at potassium metal electrodes have further been observed to travel across cells and deposit on the other electrode as a result of cross-talk,^12,13^ which could alter electrochemical behavior and lead to inaccuracies in SEI characterization. The development of a stable reference electrode for KIBs is therefore of critical importance.

A suite of alloy reference electrodes have been investigated for use in lithium- and sodium-based batteries, including the Li-Al, Li-Sn, Na-Sn, Li-Bi and Li-Au systems.^14,15^ The Li-In system has also recently received much attention as a counter/reference electrode in solid-state lithium metal battery research due to its high potential of 0.62 V vs Li^+^/Li in the In-InLi two-phase region and the high diffusivity of the InLi intermetallic phase.^16,17^ Despite this, there have been no reports investigating potassium alloys to fulfill this role in KIBs.

Here, we investigate the K-In and K-Bi alloy systems as reference electrode materials for KIBs. We synthesize and characterize bulk two-phase In-In_4_K and Bi-Bi_2_K alloys and identify Bi-Bi_2_K as the most promising. We further prove its suitability as a reference electrode by cycling graphite in three-electrode cells, and demonstrate that it results in significantly less electrolyte decomposition compared to potassium metal. This study paves the way for the implementation of Bi-Bi_2_K reference electrodes in KIB research, facilitating accurate electrochemical characterization and accelerating both electrode and electrolyte development.

Owing to the success of In-InLi reference electrodes, we investigated the K-In system. The K-In phase diagram in Figure 1a demonstrates that K and In have limited solid solubility and form several intermetallic phases,^18^ of which only the existence In_4_K has been verified experimentally^19^ and other reports suggest In_41_K_17_^20^ and In_11_K_8_^21^ are also stable. Nevertheless, In_4_K is expected to have the highest potential, so an In-10 atom %K alloy was synthesized to form a two-phase alloy, ensuring a constant potential independent of random composition fluctuations. The high volume fraction of In allows the alloy to be calendered into a foil.

**Figure 1:**
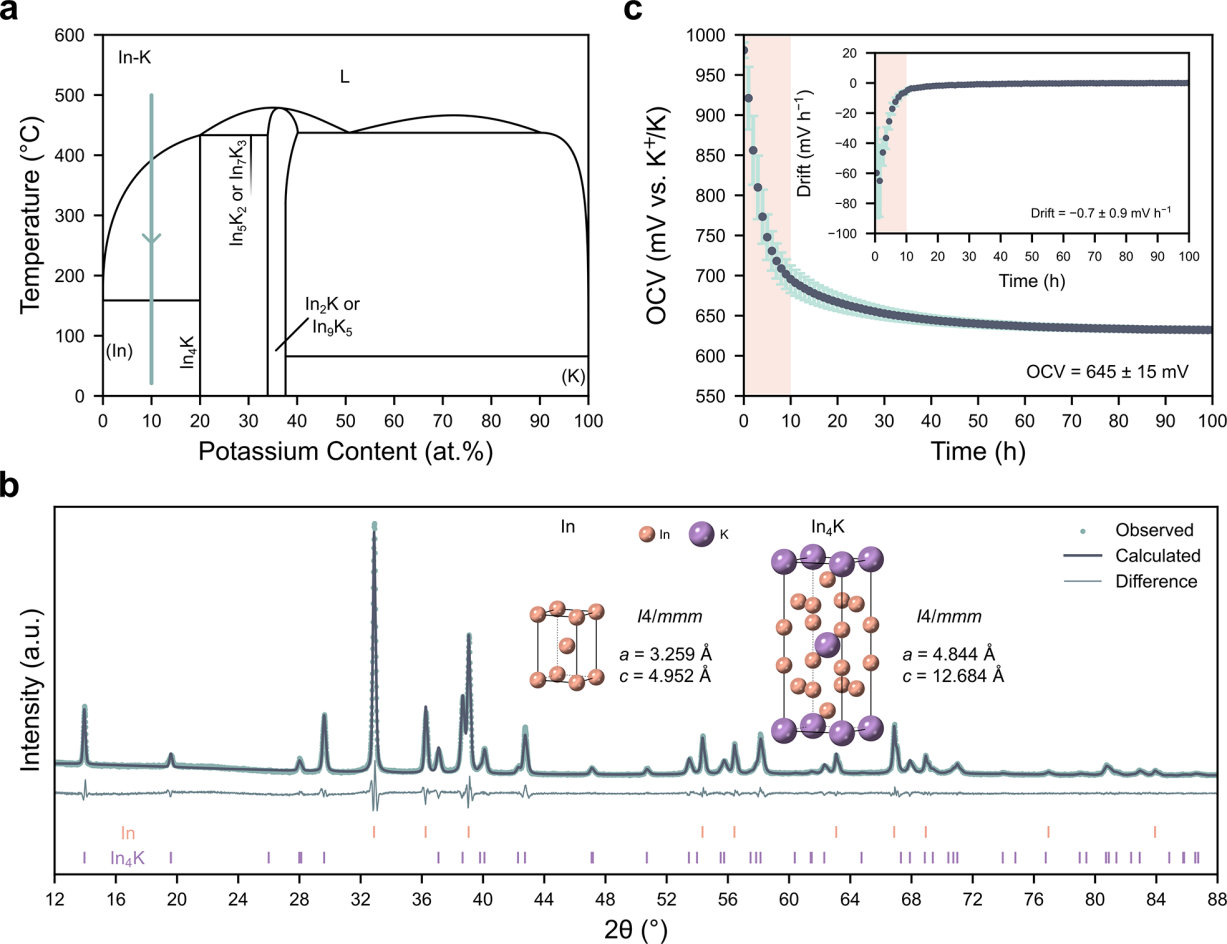
K-In system. (a) K-In phase diagram, adapted from Okamoto.^18^ The heat treatment to synthesize the target composition of In-10 atom %K is marked in blue. (b) XRD of In-10 atom %K (λ = 1.5406 Å) and Pawley refinement. The crystal structures of the identified In and In_4_K phases are shown in the inset. (c) OCV of In-10 atom %K versus potassium metal in 2.5 M KFSI-TEP at 30 °C. The voltage drift is plotted in the inset. An initial 10 h stabilization period is marked in orange and is excluded from the averages. Error bars represent the standard error in the mean determined from 7 repeat cells.

The X-ray diffraction (XRD) pattern from this material in Figure 1b confirms the presence of both In and In_4_K, with no evidence of impurity phases. The In phase has the expected tetragonal crystal structure (*I*4/*mmm*, *a* = 3.25869(7) Å, *c* = 4.95224(15) Å), and In_4_K has the BaAl_4_-type tetragonal crystal structure (*I*4/*mmm*, *a* = 4.84447(15) Å, *c* = 12.6840(4) Å), consistent with previous reports.^19^

To test the suitability of this alloy as a reference electrode it was assembled into coin cells against potassium metal and the open-circuit voltage (OCV) was tracked over 100 h at 30 °C. 2.5 M potassium bis(fluorosulfonyl)imide (KFSI) in triethyl phosphate (TEP) was used as the electrolyte. Importantly, the potassium metal electrodes were produced by a remelting, quenching, rolling, and polishing methodology,^11^ providing a sufficiently stable potential to evaluate new reference materials. The OCV of the In-In_4_K is plotted in Figure 1c, where it can be observed that the initial OCV is 980 ± 10 mV vs K^+^/K, and it relaxes to 632 ± 4 mV vs K^+^/K after 100 h, suggesting this is the true potential of K^+^/In_4_K. While the potential of 632 ± 4 mV vs K^+^/K will reduce the driving force for electrolyte reduction, it is unlikely to fall within the electrolyte electrochemical stability window, so spontaneous In_4_K oxidation is expected upon electrolyte addition. We therefore suspect the initial OCV of 980 ± 10 mV vs K^+^/K results from complete depletion of K at the surface, and the gradual decay in potential is limited by the diffusion of K to replenish the lost atoms. The OCV takes several days to stabilize, indicating sluggish diffusion kinetics, so the average potential over the last 90 h in Figure 1c (allowing for a 10 h stabilization period) is 645 ± 15 mV vs K^+^/K, with a high average potential drift of – 0.7 ± 0.9 mV h^–1^.

This long stabilization period would hinder the implementation of In-In_4_K as a reference electrode, and the sluggish kinetics of the K-In system explains the lack of available reports on its use for KIB applications and why, despite several attempts, we were unable to reversibly cycle indium metal against potassium. We therefore decided to explore alternative systems.

A range of potassium alloy systems have been explored as alternative KIB anode materials, including K-Sn, K-Pb, K-Sb and K-Bi.^5,22^ Of these, the K-Bi system appears most promising for reference electrode applications as it undergoes several two-phase reactions during cycling.^22^ Its successful use as an anode material also suggests it will not experience the same kinetic limitations as the K-In system.

To investigate the K-Bi system a thin bismuth electrode was produced. The atomic force microscopy (AFM) height map of this electrode in Figure 2a reveals that it is highly faceted and dense, with a thickness of 0.90 ± 0.14 μm (Figure S1). Bismuth was cycled against potassium metal in Figure 2b, revealing one broad plateau during the first potassiation, and three distinct plateaus during all subsequent half-cycles, consistent with previous reports.^23,24^ A 2 m KFSI-TEP electrolyte was used due to its proven performance in KIB applications and compatibility with both graphite and cathode materials,^25−27^ and all measurements were performed at 30 °C. The mechanisms taking place during cycling were investigated by Yao et al., revealing the first potassiation initiates at the Bi surface and takes place through conversion to BiK_3_ (via a Bi_2_K intermediate). This results in over 400% volume expansion, causing the electrode to fracture into small fragments which both greatly increases the area available for reaction and reduces the mechanical constraint.^28^ This enables additional reactions to take place on the following cycles, in accordance with the phase diagram in Figure 2c.^29^ Similar behavior is observed when using a 1 m KFSI in 1,2-dimethoxyethane (DME) electrolyte (Figure S2a).

**Figure 2:**
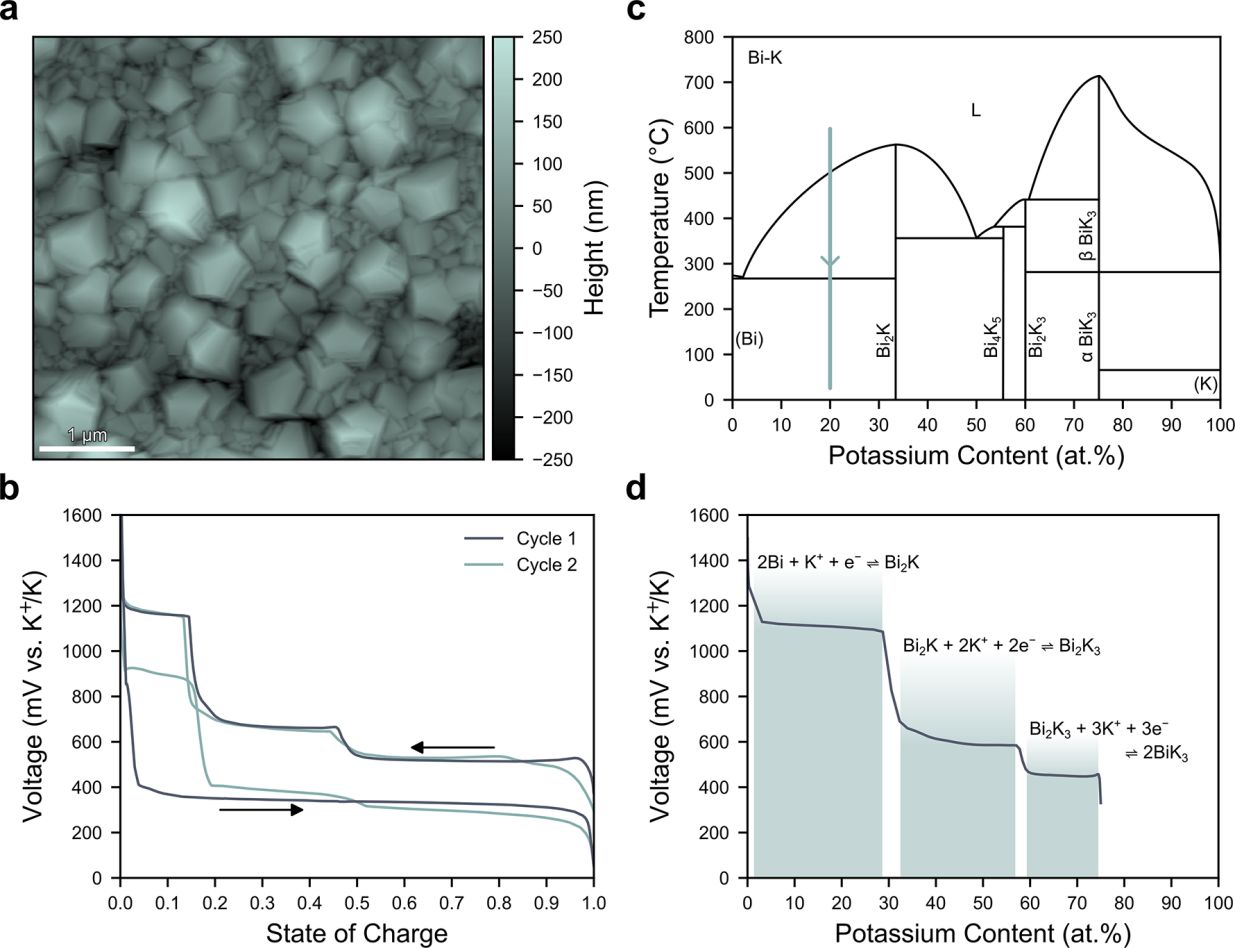
K-Bi system. (a) AFM height map of the sputtered bismuth electrode. (b) Potassiation–depotassiation curves of the bismuth electrode at C/20 in 2 m KFSI-TEP at 30 °C. (c) K-Bi phase diagram, adapted from Petric and Pelton.^29^ The heat treatment to synthesize the target composition of Bi-20 atom %K is marked in blue. (d) OCV as a function of potassium content determined by GITT during the first depotassiation in 2 m KFSI-TEP at 30 °C. The reactions taking place during each plateau are marked on the plot.

To better evaluate the potentials of the three plateaus a galvanostatic intermittent titration technique (GITT) measurement was performed during the first depotassiation to minimize the contribution of overpotentials to the observed voltages (Figure S3). The results of the GITT measurement are plotted in Figure 2d as a function of potassium content, enabling direct comparison to the phase diagram. In accordance with both the phase diagram in Figure 2c and in situ XRD measurements performed by Lei et al.,^23^ the plateaus at approximately 1100 mV, 600 mV and 450 mV vs K^+^/K correspond to 2Bi + K^+^ + e^–^ ⇌ Bi_2_K, Bi_2_K + 2K^+^ + 2e^–^ ⇌ Bi_2_K_3_ and Bi_2_K_3_ + 3K^+^ + 3e^–^ ⇌ 2BiK_3_, respectively.

Interestingly, there is evidence of an additional plateau from approximately 33–40 atom %K in Figure 2d, suggesting the conversion from Bi_2_K_3_ to Bi_2_K may take place through an additional phase. This plateau is even more evident when using 1 m KFSI-DME (Figure S2b). Similarly, an additional oxidative peak has been observed in cyclic voltammetry (CV) studies,^30,31^ but its origin has not been identified. The partial conversion of Bi_2_K_3_ to either Bi_4_K_5_ or metastable BiK^32^ could explain these observations.

Of the plateaus identified in Figure 2d, the Bi-Bi_2_K is most promising due to its high potential, greatly reducing the driving force for electrolyte reduction. A Bi-20 atom %K alloy was therefore synthesized. Unlike the In-10 atom %K, Bi-20 atom %K is hard and brittle and cannot be calendered into a foil, but it can be easily ground into a powder and polished.

To confirm the phases present in the alloy it was ground into a powder (Figure S4) and loaded into capillaries for synchrotron XRD. As shown in Figure 3a, synchrotron XRD reveals the presence of both Bi and Bi_2_K, with no impurity phases. Bi has the expected rhombohedral crystal structure (*R*3̅*m*, *a* = 4.54628(12) Å, *c* = 11.8620(6) Å) and Bi_2_K has a cubic Laves crystal structure (*Fd*3̅*m*, *a* = 9.52539(3) Å), consistent with previous reports.^23,32^

**Figure 3:**
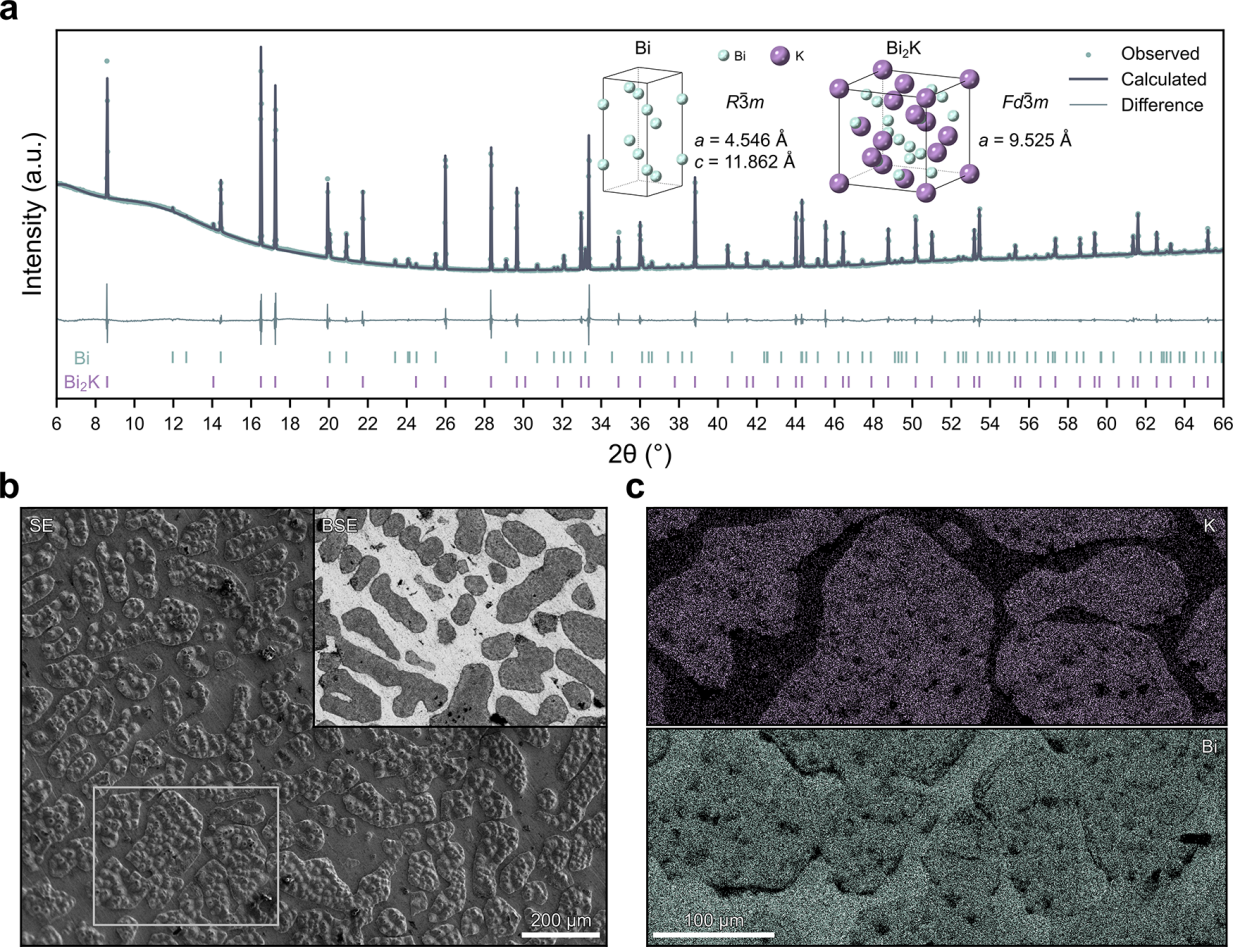
Bi-20 atom %K characterization. (a) Synchrotron XRD of Bi-20 atom %K (λ = 0.824316(2) Å) and Pawley refinement. The crystal structures of the identified Bi and Bi_2_K phases are shown in the inset. (b) SEM SE and BSE images of polished Bi-20 atom %K. (c) EDX mapping of the K (top) and Bi (bottom) distributions in the region marked by the gray box in (b).

The Bi-Bi_2_K was polished for scanning electron microscopy (SEM) characterization to explore its microstructure. The secondary electron (SE) image in Figure 3b reveals that the microstructure consists of coarse dendrites of a primary phase with primary and secondary dendrite arm spacings of approximately 500 and 100 μm, respectively. These dendrites are embedded in a matrix of a secondary phase that is preferentially removed during polishing, suggesting it is softer. The backscattered electron (BSE) image in Figure 3b provides excellent contrast between the two phases, revealing that the matrix phase has a higher average atomic number. Energy-dispersive X-ray spectroscopy (EDX) was further employed to analyze the region highlighted by the gray box in Figure 3b. As shown in Figure 3c, EDX mapping reveals the primary dendrites are rich in both K and Bi, while the matrix only contains Bi. These observations confirm that the primary dendritic phase is Bi_2_K and the matrix is Bi, in agreement with the phase diagram (Figure 2c). Analysis of the BSE image in Figure 3b reveals an alloy composition of Bi-22.2 atom %K (Figure S5), which is close to the target composition of Bi-20 atom %K. Interestingly, the phase diagram in Figure 2c suggests the existence of a eutectic reaction (L → Bi + Bi_2_K) at 271 °C and 2.5 atom %K, but there is no evidence of a eutectic structure in Figure 3b or 3c.

In addition to the two-phase Bi-Bi_2_K alloy, single-phase Bi_2_K was synthesized to investigate the properties of the Bi_2_K intermetallic in isolation. Synchrotron XRD (Figure S6a) confirms the Bi_2_K is phase pure and the crystal structure (*Fd*3̅*m*, *a* = 9.52752(5) Å) is in excellent agreement with Figure 3a. The SEM image in Figure S6b further reveals a single-phase microstructure consisting of equiaxed grains with an average diameter of approximately 500 μm and nanoindentation gives a Young’s modulus of 38.2 ± 0.3 GPa and a hardness of 1.46 ± 0.01 GPa (Figure S5c). The Young’s modulus of Bi_2_K is similar to that of Bi (33–35 GPa^33^), while its hardness is significantly higher than that of Bi (50–100 MPa^33^), as would be expected due to the highly ordered structures and directional bonding in intermetallic materials. The discrepancy in hardness between the two phases explains why Bi is preferentially polished away in Figure 3b.

Having successfully produced a pure, two-phase Bi-Bi_2_K alloy, we then evaluated its suitability as a reference electrode. To serve as a reference electrode the alloy must have a stable potential, so it was assembled into coin cells against potassium metal. As the Bi-Bi_2_K cannot be calendered into a foil on it own, Bi-Bi_2_K powder was embedded into calendered Bi granules to form an electrode (Figure S7). The OCV of these cells (at 30 °C in 2 m KFSI-TEP) was tracked over 100 h in Figure 4a, revealing that it rapidly stabilizes and, after an initial 10 h rest period, has an average potential of 1070 ± 3 mV vs K^+^/K over 90 h, consistent with Figure 2d. The potential drift is also very low, with an average of −0.1 ± 0.1 mV h^–1^ over the same period. Combining this rapid stabilization with its high potential of 1070 ± 3 mV vs K^+^/K, this makes the Bi-Bi_2_K system an attractive system for further exploration.

**Figure 4:**
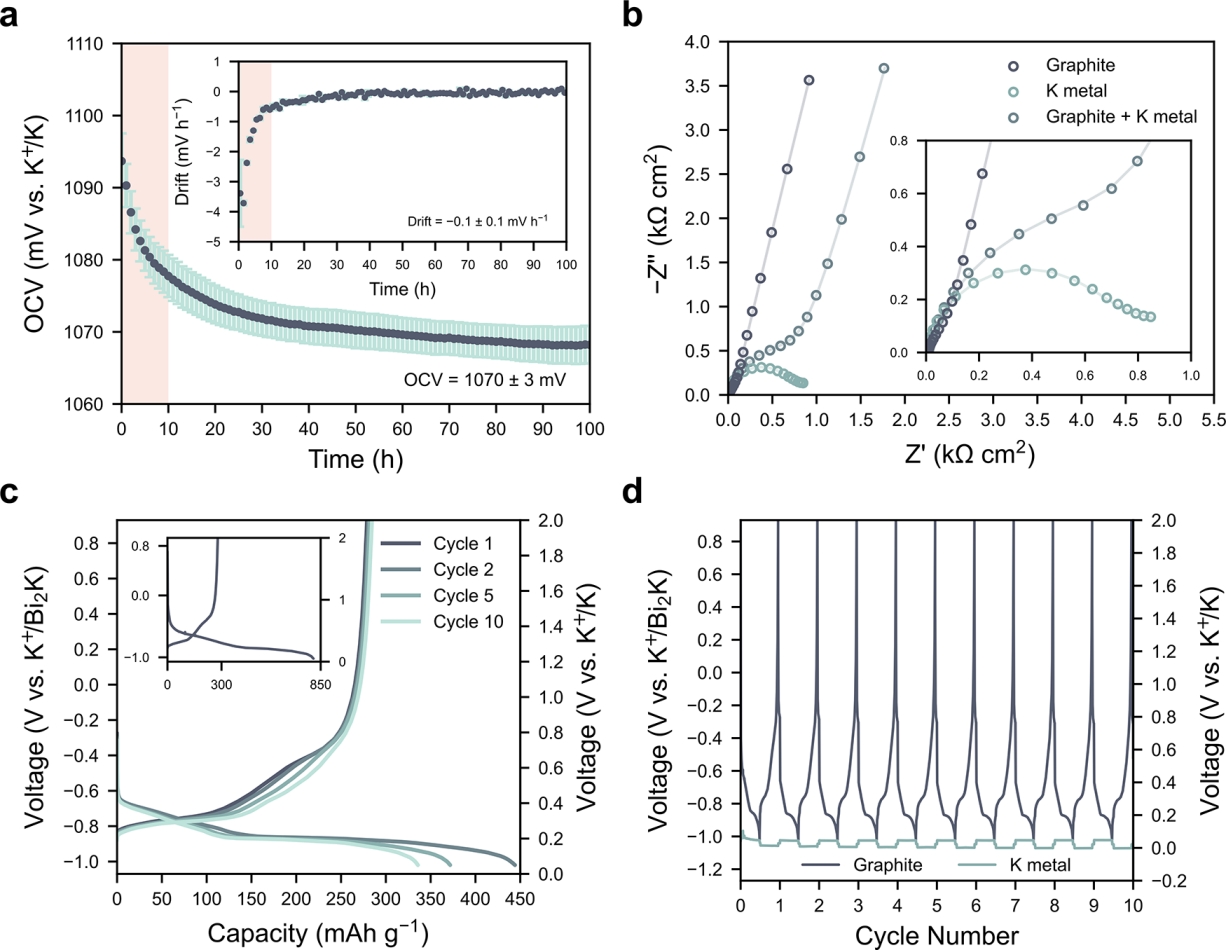
Use as a reference electrode. (a) OCV of Bi-Bi_2_K versus potassium metal. The voltage drift is plotted in the inset. An initial 10 h stabilization period is marked in orange and is excluded from the averages. Error bars represent the standard error in the mean determined from 3 repeat cells. (b) Impedance spectra from the graphite and potassium metal electrodes, plus their sum, after 24 h at OCV. (c) Graphite cycling at C/10. The full first cycle is shown in the inset. (d) Voltage profiles of both the graphite and potassium metal electrodes during the first 10 cycles. All measurements were performed in 2 m KFSI-TEP at 30 °C.

To further test the use of Bi-Bi_2_K as a reference electrode, it is necessary to assemble three-electrode cells to evaluate its performance in a realistic use case. We therefore constructed ECC-ref cells (EL-cell) with a graphite working electrode and a potassium metal counter electrode, using a small needle of the as-cast Bi-Bi_2_K alloy as the reference electrode. A 2 m KFSI-TEP electrolyte was again used and measurements were performed at 30 °C.

Figure 4b shows the electrochemical impedance spectroscopy (EIS) spectra gathered from the three-electrode cell after a 24 h rest period at OCV, demonstrating that the impedance contributions from the graphite and the potassium metal can be separated successfully. Fitting of the impedance spectra (Figure S8 and Tables S1 and S2) reveals a high total interfacial resistance of 1050 ± 70 Ω cm^2^ for the potassium metal electrode, demonstrating that the potassium metal counter electrode would significantly impact the impedance response in two-electrode cells, as evident when the individual impedance spectra are summed together in Figure 4b. This highlights the importance of three-electrode cells for accurate impedance characterization. Without a suitable reference electrode, such characterization would require the ex-situ preparation of symmetric cells,^15^ preventing operando measurements.

Following this rest period the graphite was cycled between –1.02 and 0.93 V vs K^+^/Bi_2_K (0.05–2 V vs K^+^/K) at C/10, as shown in Figure 4c. The first cycle potassiation capacity of 810 mAh g^–1^ is much higher than the theoretical capacity of 279 mAh g^–1^, while the capacity extracted during the first depotassiation is close to theoretical. This high excess capacity on the first cycle is consistent with previous reports,^25^ and is attributed to irreversible electrolyte reduction and SEI formation, which may be exacerbated by the large area of exposed metallic cell components and the low loading (0.5 mg cm^–2^) used here. Therefore, although it decreases with increasing number of cycles, the potassiation capacity is consistently above 279 mAh g^–1^ in Figure 4c, while the depotassiation capacity remains close to theoretical. Nevertheless, this serves as a successful proof-of-concept for the use of Bi-Bi_2_K, proving that potassium alloy materials can serve as reliable reference electrodes for KIBs.

Figure 4d further shows the voltage profiles of both the graphite and potassium metal during cycling, demonstrating that the potentials of working and counter electrodes can be accurately separated and there is negligible reference drift. Stable cycling was maintained for several weeks with no evidence of reference degradation (Figure S9). This advancement will enable full-cell KIB characterization in three-electrode cells without the need for potassium metal, eliminating any influence it may have on electrochemical performance. Additionally, the stable potential of Bi-Bi_2_K would allow the use of alternative counter electrodes that do not have a constant potential, further eliminating the need to utilize highly reactive potassium metal in half-cell studies.

So far we have proven that Bi-Bi_2_K has a stable, well-defined potential, satisfying one of the key reference electrode criteria. However, another key requirement is electrochemical stability to ensure that the reference will not have significant impact on the system under study.

To explore the potential at which electrolyte reduction reactions occur, cyclic voltammetry (CV) was performed to sweep an aluminum working electrode between 1800 and 50 mV vs K^+^/K at a scan rate of 100 μV s^–1^ (Figure 5a). During the first cathodic sweep there are several broad reductive waves indicative of electrolyte reduction, with apparent peaks at approximately 850 and 600 mV vs K^+^/K followed by further reduction as the potential decreases. No corresponding oxidative peaks appear in the following anodic sweep and all subsequent cycles display pseudocapacitive behavior with reduced current densities during the cathodic sweeps. This implies that the electrolyte reduction during the first cycle is irreversible and it forms a passivating SEI, preventing further decomposition.^34^ Similar behavior is observed when using a 1 m KFSI-DME electrolyte (Figure S10), suggesting FSI^–^ is the main species undergoing reduction, rather than the solvent. Importantly, the potential of Bi-Bi_2_K (1070 ± 3 mV vs K^+^/K) is higher than the potential of electrolyte reduction, implying it is stable in the electrolyte systems considered here.

**Figure 5:**
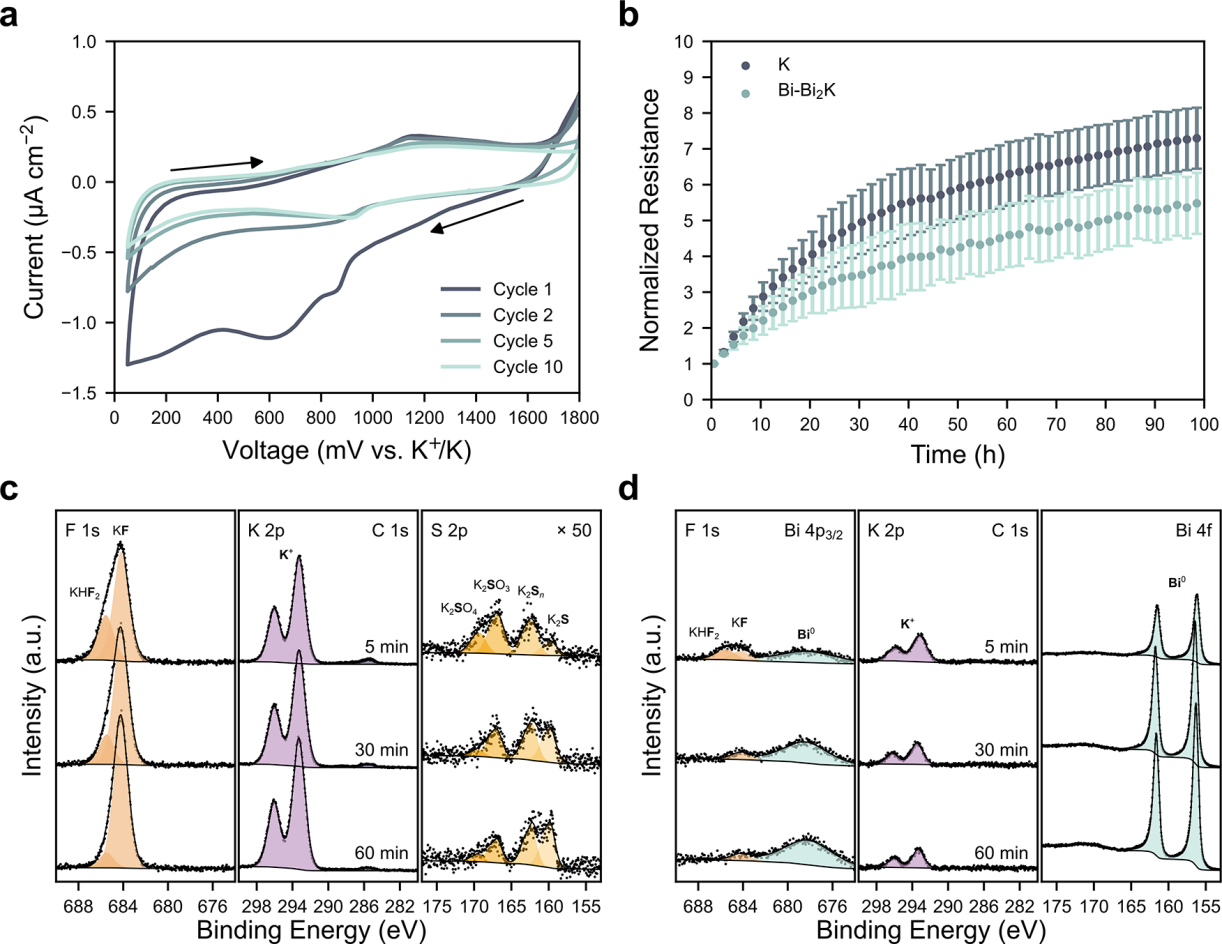
Electrochemical stability and SEI formation. (a) CV curves of an aluminum electrode at 100 μV s^–1^ in 2 m KFSI-TEP at 30 °C. (b) Interfacial resistance evolution from K∥K and Bi-Bi_2_K∥Bi-Bi_2_K cells at OCV, normalized by the resistance after 30 min, in 2 m KFSI-TEP at 30 °C. Error bars represent the standard error in the mean from at least 2 repeat cells. (c) F 1s, K 2p, C 1s, and S 2p XPS spectra from depth profiles on the SEI formed on potassium metal. (d) F 1s, Bi 4p_3/2_, K 2p, C 1s, and Bi 4f XPS spectra from depth profiles on the SEI formed on Bi-Bi_2_K. Both SEI samples were formed by submersion in 2 m KFSI-TEP for 20 h at 30 °C. Spectra measured after 5, 30 and 60 min of sputtering are presented and consistent *y*-scales are used in (c) and (d), except the S 2p spectra in (c) are scaled by a factor of 50 relative to the Bi 4f spectra in (d).

While other electrolyte systems and electrolyte additives may undergo reduction at higher potentials,^35,36^ Bi-Bi_2_K will still be far less reactive than potassium metal. This reduced reactivity is evident when storing and disposing of the Bi-Bi_2_K alloy. Potassium metal readily reacts with atmospheric gases, even in a well-controlled glovebox atmosphere, and it reacts violently with water, making its storage and disposal challenging. In contrast, Bi-Bi_2_K maintained its shiny appearance and purity even after several months of glovebox storage. It also does not react violently with water, offering enhanced safety compared to potassium metal.

To further compare the electrochemical stability of Bi-Bi_2_K to potassium metal, symmetric coin cells were assembled and EIS was performed over 100 h at OCV. As evident in Figure S11, the interfacial resistance of the potassium cells increases continuously during rest, increasing by a factor of approximately 7 over the 100 h (Figure 5b). Similarly, the corresponding interfacial resistance of the Bi-Bi_2_K cells (Figure S12) increases by a factor of approximately 5 over the same period (Figure 5b). The interfacial resistance in symmetric alkali metal cells is often assumed to be dominated by the resistance of cation migration through the SEI,^37,38^ and an increasing resistance is attributed to SEI growth,^39^ suggesting the SEI continuously increases in thickness at OCV in both systems. However, other theories suggest the SEI densifies over time and the increasing resistance is a result of a decreasing ionic conductivity.^40^ Due to the very different potentials of potassium and Bi-Bi_2_K they are likely to form SEIs with different properties, requiring further SEI characterization to interpret these trends in resistance.

X-ray photoelectron spectroscopy (XPS) depth profiling was therefore performed on potassium metal (Figure S13) and Bi-Bi_2_K (Figure S14) samples that were submerged in 2 m KFSI-TEP electrolyte for 20 h at 30 °C, followed by rinsing with TEP to remove residual salt. Examination of the XPS spectra from the potassium sample reveals that it is enriched in elements from salt decomposition and fluorine is especially prevalent. Two distinct fluorine environments are evident in the F 1s spectra in Figure 5c. The peak at 684.2 eV is attributed to KF^41^ and was utilized for charge referencing, while the peak at 685.5 eV may be consistent with KHF_2_, which is supported by measurements performed on reference samples (Figure S15). KF is already the majority F-containing species after 5 min of sputtering, and its proportion increases with sputtering time. The K 2p region displays an intense doublet peak that cannot be deconvolved, preventing meaningful identification of species based on this region alone, while the C 1s peaks have low intensity, confirming that the SEI is primarily inorganic and the solvent does not significantly contribute to the SEI, as suspected from the CV in Figure 5a. Four unique environments can be identified from the S 2p spectra, which could be attributed to K_2_SO_4_, K_2_SO_3_, K_2_S_*n*_ and K_2_S at approximately 169.0, 166.6, 162.1 and 159.7 eV, respectively.^25,42,43^ Similar to the fluorine-containing species, the sulfur-containing species become more reduced as the sputtering time increases. This could result from the fact that the driving force for reduction is greatest closest to the potassium surface, so the SEI is composed of more reduced species there.^44^ Alternatively, it has been reported that the argon sputtering used for XPS depth profiling can cause sample damage, which may contribute to the changes observed with sputtering time.^45^ Nevertheless, the SEI formed on potassium metal is rich in salt decomposition species throughout the entire examined depth (Figure S16), suggesting it is relatively thick.

In contrast, the SEI formed on the Bi-Bi_2_K appears to be thinner and less fluorine rich (Figure S17). Like the potassium metal SEI, KHF_2_ and KF are identified in the F 1s spectra in Figure 5d, but their intensities are lower and they decrease with increasing sputtering time. The K 2p and C 1s peaks also have lower intensity. After 5 min of sputtering there is no evidence of S 2p peaks, and instead a doublet peak corresponding to metallic Bi is present at 156.4 eV. The intensity of this peak initially grows with sputtering time and then reaches a plateau after approximately 20 min of sputtering, suggesting the SEI has been etched away. Beyond this point bismuth and potassium make up over 70% of all elements, as shown in Figure S17. Therefore, this suggests that, despite the normalized interfacial resistances increasing by similar factors in Figure 5b, Bi-Bi_2_K results in significantly less electrolyte decomposition than potassium metal overall, confirming that Bi-Bi_2_K satisfies another key reference electrode requirement.

In conclusion, we have investigated the K-In and K-Bi alloy systems for use as reference electrode materials for potassium-ion batteries (KIBs). We successfully synthesized pure two-phase In-In_4_K and Bi-Bi_2_K alloys, which displayed potentials of 0.63 and 1.07 V vs K^+^/K, respectively. While we reveal that the use of In-In_4_K is hindered by a kinetic limitation, we demonstrate than Bi-Bi_2_K stabilizes rapidly, enabling its implementation as a KIB reference electrode. By cycling graphite against potassium metal in three-electrode cells we prove that Bi-Bi_2_K provides a stable potential with negligible drift, even after several weeks. We further show that its high potential results in enhanced safety and significantly less electrolyte decomposition compared to potassium metal. This study paves the way for future full- and half-cell KIB studies without the need for potassium metal, eliminating any influence it has on electrochemical performance and solid electrolyte interphase formation.

## Data Availability

All the experimental data gathered in this study are available in the Zenodo database under a Creative Commons Attribution 4.0 International License (10.5281/zenodo.13327086).

## Supporting Information Available

Experimental methods and Figures S1–S17 and Tables S1 and S2, including Bi film thickness measurement, 1 m KFSI-DME electrochemistry, SEM images of Bi-Bi_2_K powder and electrodes, Bi-Bi_2_K phase quantification, Bi_2_K characterization, full impedance spectra and fitting details, full XPS spectra and quantification, and XPS spectra from KFSI, KHF_2_, and KF reference materials (PDF)
