# Supplementary material for: Potassium Alloy Reference Electrodes for Potassium-Ion Batteries: The K-In and K-Bi Systems
Source: ACS Mater Lett. 2024 Aug 30;6(10):4498–506. doi: 10.1021/acsmaterialslett.4c01219 (PMC11462602; doi:10.1021/acsmaterialslett.4c01219)
Supplement: Supplementary file 1 — tz4c01219_si_001.pdf [file tz4c01219_si_001.pdf]

Supporting Information

Potassium Alloy Reference Electrodes for Potassium-Ion Batteries: The K-In and K-Bi Systems

Ben Jagger[1], Jack Aspinall[1], Souhardh Kotakadi[1], John Cattermull[1,2], Shobhan Dhir[1], and Mauro Pasta[1*]

[1]Department of Materials, University of Oxford, Oxford OX1 3PH, UK
[2]Inorganic Chemistry Laboratory, Department of Chemistry, University of Oxford, Oxford OX1 3QR, UK
[*]Corresponding author: mauro.pasta@materials.ox.ac.uk

Contents

Experimental Methods **1**
Potassium Metal Cleaning 1
Alloy Synthesis 1
X-ray Diffraction 1
Electrode Preparation 2
Electrolyte Preparation 2
Electrochemical Measurements 3
Atomic Force Microscopy 4
Electron Microscopy 4
Nanoindentation 4
X-ray Photoelectron Spectroscopy 4

Supporting Figures **6**

Supporting Tables **19**

Supporting References **20**

Experimental Methods

Unless otherwise stated, all experiments were performed in argon-filled MBraun gloveboxes with O_2 and H_2O concentrations below 0.1 ppm and all equipment was cleaned and dried overnight under vacuum at 70°C before being brought into the glovebox.

Potassium Metal Cleaning

Before use, potassium metal (chunks, 98%, Sigma-Aldrich) was processed according to a previous report.[1] The potassium was melted in a beaker on a hot plate, a spatula was used to remove the dross, and the molten potassium was quenched into clean mineral oil before being transferred into hexane (95% anhydrous, Sigma-Aldrich) that had been dried over 3 Å molecular sieves for storage.

Alloy Synthesis

All potassium alloys were synthesized by weighing out cleaned potassium metal and either indium (pieces, 99.99%, Alfa Aesar) or bismuth (granules, >99.99%, Sigma-Aldrich) in the desired atomic ratio and adding them to a custom-designed stainless steel crucible[2] lined with molybdenum foil (Goodfellow). The mixtures were then heated in a box furnace (MTI KSL-1200X-J-UL or MTI KSL-1100X).

The In-In_4K (In-10at.%K) alloy was heated to 500°C over 100 min and then held for 2 h. The crucible was removed from the furnace and the melt was stirred with a stainless steel spatula, before being returned to the furnace for 1 h. The molybdenum foil and the melt were then removed from the crucible and quenched in mineral oil.

The Bi-Bi_2K (Bi-20at.%K) alloy and the single-phase Bi_2K (Bi-33.3at.%K) were heated to 600°C over 90 min and then held for 1 h, stirring with a stainless steel spatula once molten. They were then slow-cooled in the furnace.

X-ray Diffraction

For X-ray diffraction (XRD) the In-In_4K was rolled by hand to produce a flat foil. This was measured using a Rigaku Miniflex XRD contained within a nitrogen-filled glovebox at room temperature. A $Cu_{K\alpha}$ source (λ = 1.5406 Å) was used, with a nickel filter used to remove $Cu_{K\beta}$ radiation.

The Bi-Bi_2K and Bi_2K were ground by hand in an agate mortar and sealed into glass capillaries for XRD. Synchrotron XRD measurements were performed on the I11 beamline of the Diamond Light Source, UK, operating with an X-ray wavelength of 0.824316(2) Å. A position sensitive detector (PSD) was used to collect diffraction patterns in capillary transmission geometry. Room temperature X-ray diffraction patterns were collected using the Mythen-2 PSD, two data collections of 20 s each were taken at angles 0.25° apart, then summed to account for gaps in the detector coverage.

Pawley refinements were carried out using TOPAS-Academic software[3] to determine the crystal structures and lattice parameters of the phases present. Pawley refinements were made using models from literature,[4,5] and a Thompson-Cox-Hasting pseudo-Voigt (TCHZ) peak shape function with peak intensities allowed to freely refine. The data from the In-In_4K presented in Figure 1b was shifted down by $2\theta = 0.368°$ to correct for zero error.

Electrode Preparation

The clean potassium chunks were rolled by hand between two sheets of weighing paper (grade 2122, Whatman), using additional hexane to prevent sticking, to a thickness of approximately 500 µm. The potassium foil was then punched into 10 mm diameter electrodes using a wad punch. Immediately before cell assembly the surface of the potassium electrodes was polished with a plastic blade, producing a mirror-like finish.

The In-In_4K electrodes were prepared by calendering the as-cast alloy down to a thickness of 300 µm and then punching 8 mm diameter electrodes with a wad punch.

The 10 mm diameter bismuth film electrodes were deposited onto masked stainless steel spacers (304SS, MTI) by radio-frequency (RF) magnetron sputtering in an MBraun MB-Evap physical vapor deposition system. A 50 mm diameter bismuth sputtering target (BI00-ST-000100, Goodfellow) was used with a sputtering power of 28 W. The sputtering chamber was pumped down to a pressure of 5×10^{-5} mbar prior to sputtering. Argon gas was used as the sputtering gas and was injected at a flow rate of 10 SCCM. Prior to deposition the bismuth target was cleaned of impurities by pre-sputtering, with the substrate shutter closed, for 2 min at the same power. During deposition the substrate was rotated at a rate of 33 rpm. The sputtering duration was 2 h.

Bi-Bi_2K electrodes for two-electrode studies were prepared by calendering bismuth granules (>99.99%, Sigma-Aldrich) down to a thickness of 350 µm, adding Bi-Bi_2K powder (ground by hand in an agate mortar, Figure S4) on top, and then calendering to a thickness of 300 µm (Figure S7). Due to the brittle nature of bismuth, it was not possible to produce a large, uniform film, so these electrodes had approximate dimensions of 4 mm × 4 mm.

Bi-Bi_2K electrodes for three-electrode studies were prepared by breaking off small needles of the as-cast alloy, with approximate dimensions of 500 µm × 500 µm × 2 mm.

Graphite electrodes were prepared by mixing 92 wt.% graphite flakes (SGP5, SEC Carbon) with 8 wt.% sodium carboxymethyl cellulose (CMC) binder (Sigma-Aldrich), using ultrapure deionized water as the solvent. This slurry was cast onto a carbon-coated aluminum current collector (18 µm thick, MTI) and dried in air at room temperature for 24 h, and then under vacuum at 100°C for 24 h. The casting was then punched into 10 mm diameter electrodes, with a loading of approximately 0.5 mg cm^{-2}.

Aluminum electrodes for cyclic voltammetry studies were prepared by cleaning and drying aluminum foil before punching it into 10 mm diameter disks.

Electrolyte Preparation

Prior to preparing electrolytes, potassium bis(fluorosulfonyl)imide (KFSI, 99.9%, Solvionic) was dried under vacuum at 100°C for at least 48 h, triethyl phosphate (TEP, 99.8%+, Sigma-Aldrich) was dried over potassium metal strips for at least 1 week, and 1,2-dimethoxyethane (DME, 99.5% anhydrous, Sigma-Aldrich) was dried over 3 Å molecular sieves.

Electrolytes were prepared gravimetrically by stirring together the desired masses of salt and solvent. The water content of the electrolytes was measured by Karl Fischer titration and recorded to be below 5 ppm.

Electrochemical Measurements

All electrochemical measurements were performed at 30°C (± 0.3°C) in either a Binder Oven or an ESPEC SH-262 temperature and humidity chamber. Symmetric coin cells and three-electrode cells were measured using a Biologic VMP3 potentiostat, and a Biologic BCS battery cycler was used in all other cases.

Two-electrode CR2032 coin cells (304SS, MTI) were assembled with the electrodes described above using a wave spring (304SS, MTI), a single spacer (500 µm thick, 304SS, MTI) and a single glass microfiber separator (grade GF/F, Whatman). 200 µL of electrolyte was used. Unless otherwise stated, all cells were allowed to stabilize for at least 10 h at OCV before testing.

K||In-In_4K cells utilized 2.5 M KFSI-TEP and were measured at OCV for 100 h.

K||Bi cells were cycled at approximately C/20 (18.78 µA cm^{-2}) between 0.05–1.5 V vs. K^+/K, with a 1 h rest at OCV between half cycles. For GITT characterization, the cells were first potassiated at C/20 (18.78 µA cm^{-2}), followed by repeated 15 min C/20 current pulses and 2 h OCV periods until the upper cutoff voltage was reached during depotassiation (Figures S2c and S3). These measurements were performed with both 2 m KFSI-TEP and 1 m KFSI-DME.

K||Bi-Bi_2K cells utilized 2 m KFSI-TEP and were measured at OCV for 100 h.

Cyclic voltammetry (CV) was performed on K||Al cells by cycling between 1.8 V and 0.05 V vs. K^+/K with a scan rate of 100 µV s^{-1}. Both 2 m KFSI-TEP and 1 m KFSI-DME were used.

Electrochemical impedance spectroscopy (EIS) was performed on symmetric K||K and Bi-Bi_2K||Bi-Bi_2K cells every 30 min for 100 h at OCV. A frequency range of 200 kHz–500 mHz (6 points per decade) was used with a voltage amplitude of 10 mV. 2 m KFSI-TEP was used. Impedance spectra were fitted using the impedance.py Python package,[6] as described in Figures S11 and S12. The interfacial resistances in Figure 5b were normalized by the resistance after 30 min at rest to ensure temperature equilibration.

EL-cell ECC-Ref three-electrode cells were constructed using a potassium metal counter electrode, graphite working electrode and Bi-Bi_2K reference electrode. A single glass microfiber separator (grade GF/F, Whatman) was used, with 600 µL of 2 m KFSI-TEP electrolyte. The cells were allowed to rest for 24 h at OCV, and EIS was performed every 30 min (200 kHz–500 mHz, 6 points per decade, 10 mV voltage amplitude). The impedance spectra after 24 h were fit using the impedance.py Python package,[6] as described in Figure S8 and Tables S1 and S2. The graphite electrodes were then cycled at C/10 (13.82 µA cm^{-2} for the cell presented in Figures 4c, 4d and S9) between −1.02 V and 0.93 V vs. K^+/Bi_2K, corresponding to 0.05–2 V vs. K^+/K. The cell was allowed to rest at OCV for 1 h between half cycles.

Atomic Force Microscopy

Atomic force microscopy (AFM) was performed with a Bruker Dimension Icon system. The height map in Figure 2a was gathered in the ScanAsyst imaging mode with a ScanAsyst-Air probe (Bruker) at a scan rate of 1 Hz (512 px × 512 px). To determine the thickness of the bismuth film it was scratched with the tip of stainless steel tweezers to produce a sharp step (Figure S1). This was then imaged in contact mode using an SNL-B probe (Bruker) at a scan rate of 0.5 Hz (256 px × 256 px). Gwyddion software was used for data analysis.[7]

Electron Microscopy

Samples for scanning electron microscopy (SEM) were polished using silicon carbide grinding papers with grits from 800–4000, followed by final polishing with 1 µm and 0.5 µm diamond polishing pads (Kemet).

SEM and energy-dispersive X-ray spectroscopy (EDX) were performed within a TESCAN MIRA-3 FEG-SEM equipped with an Oxford Instruments EDX detector. SEM imaging and EDX mapping were both performed with a beam voltage of 10 kV. The SEM opens into an MBraun glovebox, preventing air exposure. ImageJ software was used to quantify phase fractions from backscattered electron (BSE) data (Figure S5).[8]

Nanoindentation

The Bi_2K sample for nanoindentation was mounted on an aluminum SEM stub with two-part Araldite epoxy and was polished in the same way as the SEM samples.

Nanoindentation was performed using a Bruker PI 89 nanoindenter contained within the TESCAN MIRA-3 FEG-SEM. A diamond Berkovich indentation tip (Hysitron) was used. 29 indents were performed, where the load was linearly increased over 5 s to a maximum load of 200 mN, held for 2 s, then linearly unloaded over 5 s (Figure S6c). Young's modulus and hardness were calculated from indentation moduli using diamond values of $E = 1220$ GPa, $\nu = 0.07$.[9]

X-ray Photoelectron Spectroscopy

Samples for X-ray photoelectron spectroscopy (XPS) were prepared by submerging potassium metal and Bi-Bi_2K electrodes in 1 mL of 2 m KFSI-TEP each. These were then kept in a glovebox antechamber heated to 30°C for 20 h before they were removed from the electrolyte and rinsed three times each with 200 µL of pure TEP to remove residual salt. The excess TEP was then removed with tissue, taking care not to touch the electrode surface. Samples were then immediately transferred to the XPS intro chamber using a vacuum transfer vessel (ULVAC PHI) to prevent air exposure.

XPS was performed with an ULVAC PHI Versaprobe III XPS system generating monochromatic $Al_{K\alpha}$ X-rays (1486.6 eV, 15 kV anode voltage, 25 W beam power) under ultrahigh vacuum (UHV) conditions (10^{-7}–10^{-6} Pa). A 500 µm × 500 µm area of each sample was analyzed. Survey spectra were gathered at a pass energy of 224 eV, and 55 eV was used for core-level spectra. In-built electron and low-energy Ar^+ sources were utilized for neutralization. Depth profiling was achieved with consecutive XPS analysis and Ar^+ sputtering (2 kV, 3 mm × 3 mm) for a total of 60 min. Acquired spectra were fitted with Gaussian-Lorentzian product lineshapes (with an asymmetric lineshape necessary to fit the Bi^0 Bi 4f doublet peak) using CasaXPS software.[10] Due to the lack of an intense adventitious carbon peak, all spectra were charge referenced to the KF F 1s peak at 684.2 eV.[11] Atomic percentages were estimated using Scofield relative sensitivity factors (RSFs).

Reference F 1s XPS spectra were gathered from KFSI (99.9%, Solvionic), KHF_2 (99%, Sigma-Aldrich) and KF ($\geq$99.9%, Sigma-Aldrich). No Ar^+ sputtering was performed prior to analysis.

Supporting Figures

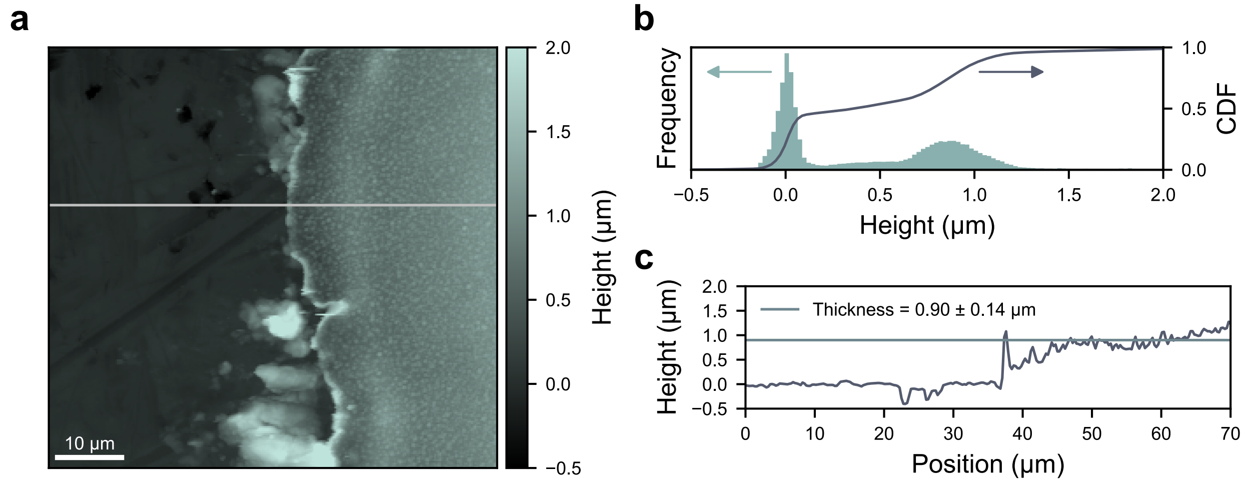

Figure S1. Bismuth film thickness measurement. (a) AFM height map across a step created through the bismuth film. (b) Histogram of heights in (a) and corresponding cumulative distribution function (CDF), showing peaks corresponding to both the substrate and the film. (c) Height across the 1D section marked in (a) compared with the average film thickness.

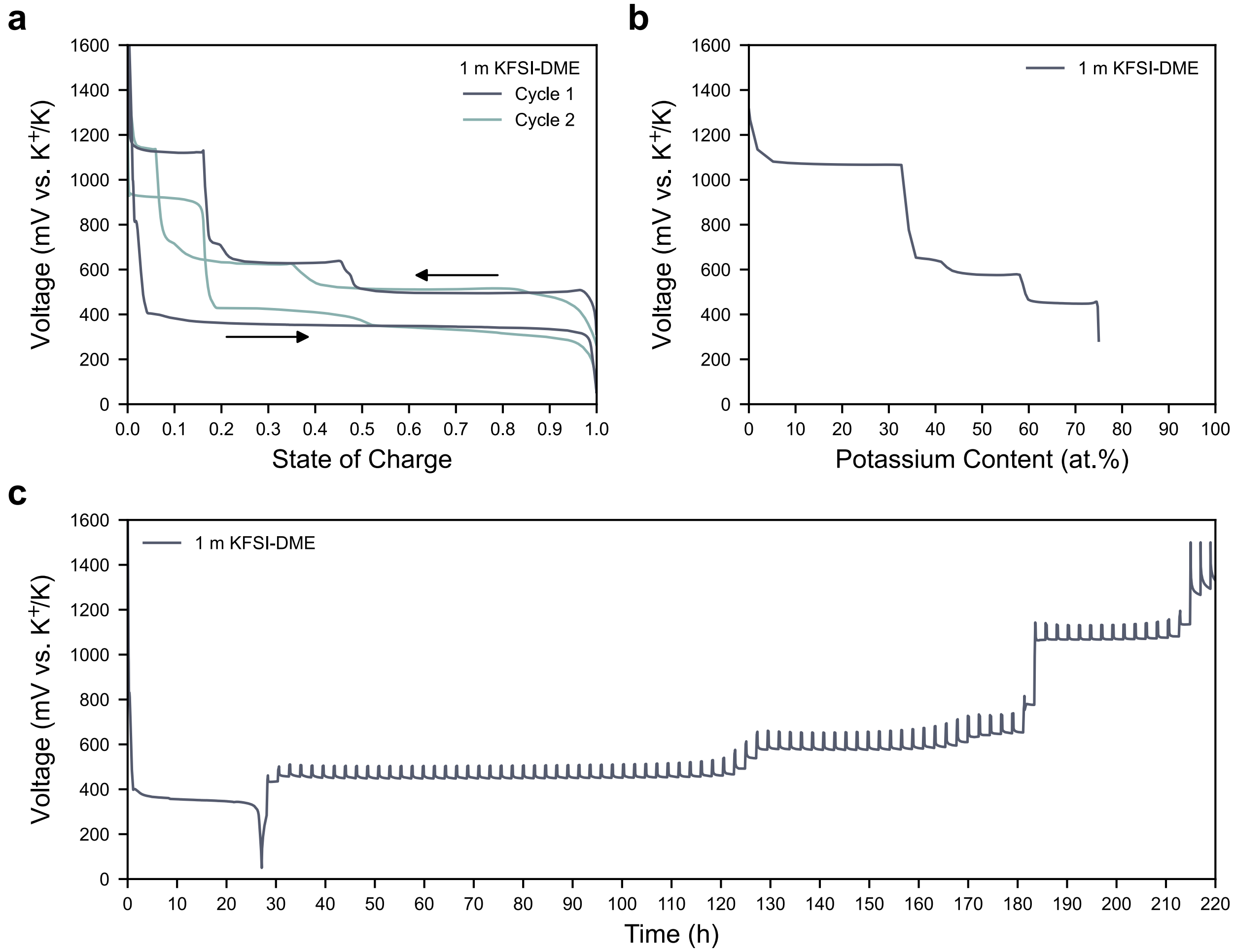

Figure S2. Bi||K cells in 1 m KFSI-DME (a) Bismuth cycling against potassium at approximately C/20. (b) OCV as a function of potassium content. (c) Raw GITT data gathered during the first depotassiation. All measurements were performed at 30°C.

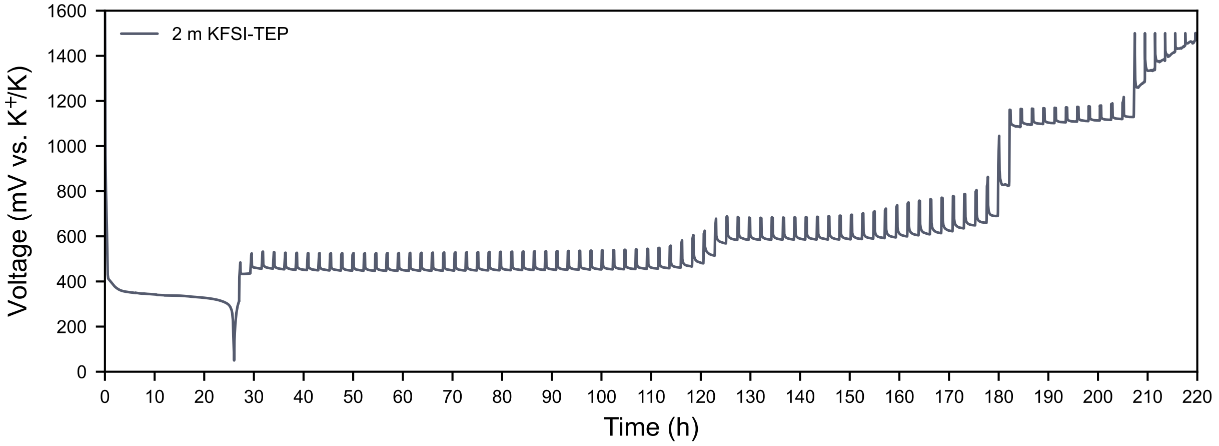

Figure S3. Bi||K GITT in 2 m KFSI-TEP. Raw GITT data gathered during the first depotassiation at 30°C.

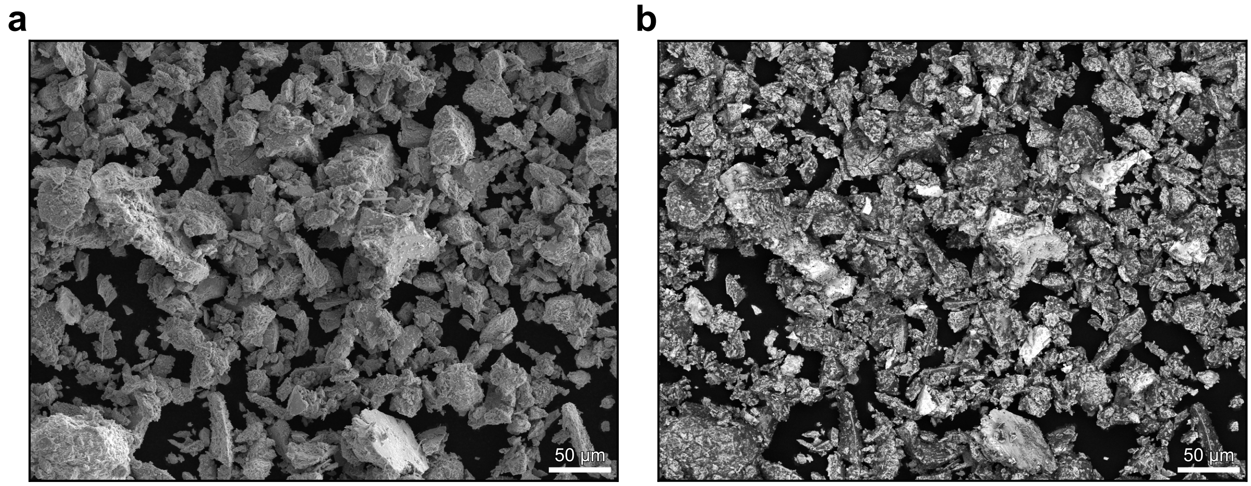

Figure S4. Bi-Bi_2K powder SEM. (a) Secondary electron (SE) image. (b) Backscattered electron (BSE) image, revealing contrast between the two phases.

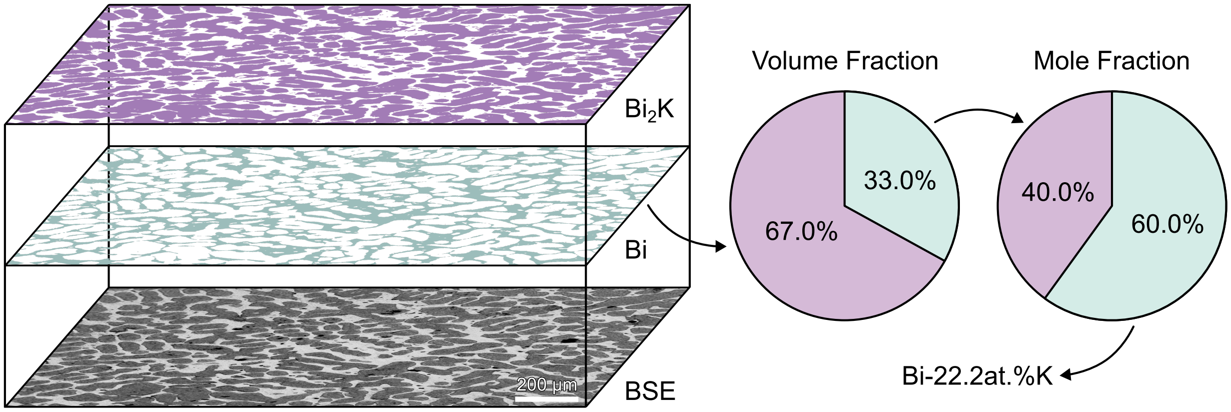

Figure S5. Bi-Bi$_2$K phase quantification. Backscattered electron (BSE) image and extracted Bi and Bi$_2$K phase distributions used to calculate phase volume and mole fractions, as well as alloy composition. The BSE image was analyzed using ImageJ.[8]

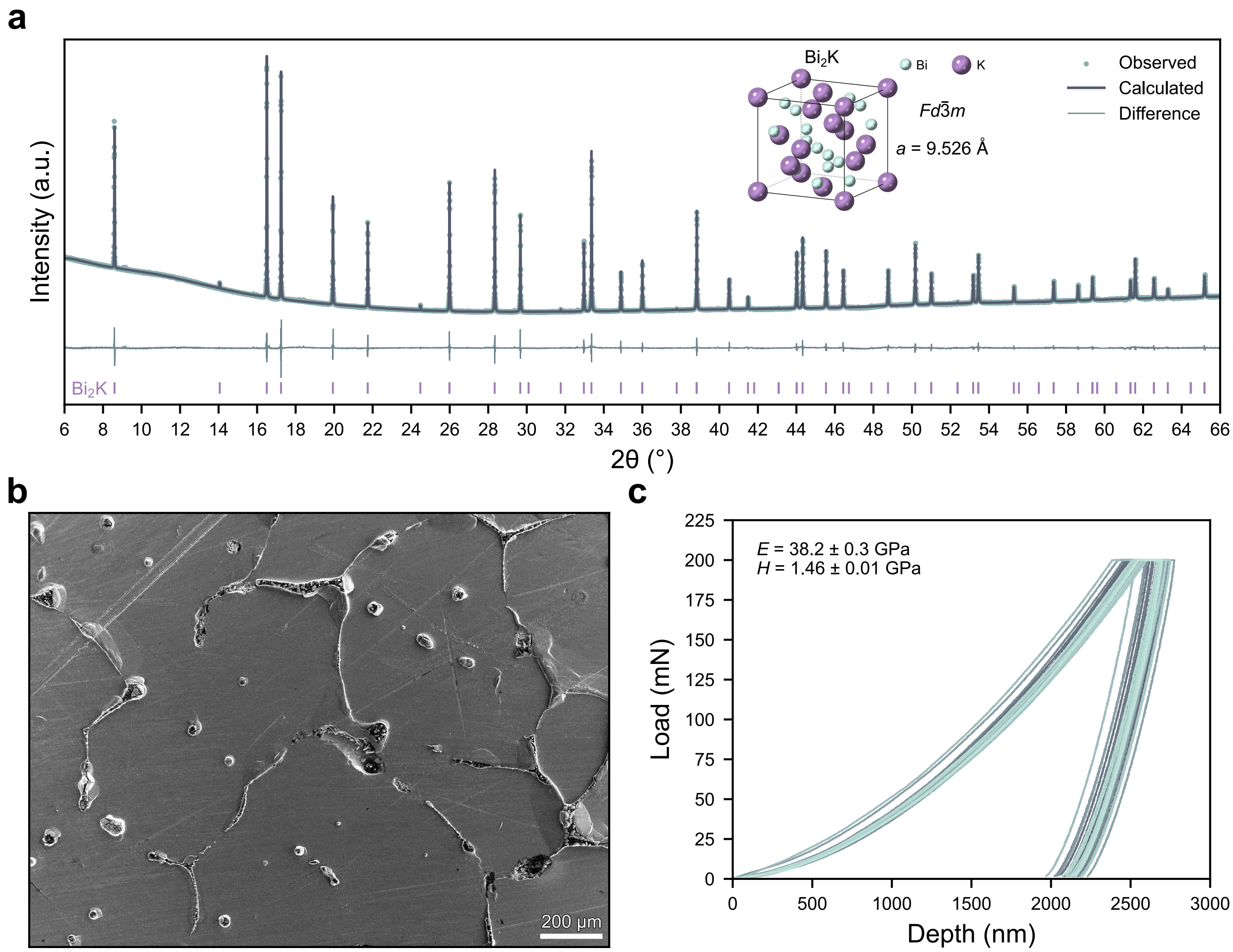

Figure S6. Bi_2K characterization. (a) Synchrotron XRD (λ = 0.824316(2) Å) and Pawley refinement. The crystal structure is shown in the inset. (b) SEM secondary electron (SE) image of polished Bi_2K. (c) Nanoindentation curves used to measure the Young's modulus, E, and hardness, H. 29 nanoindentations were performed.

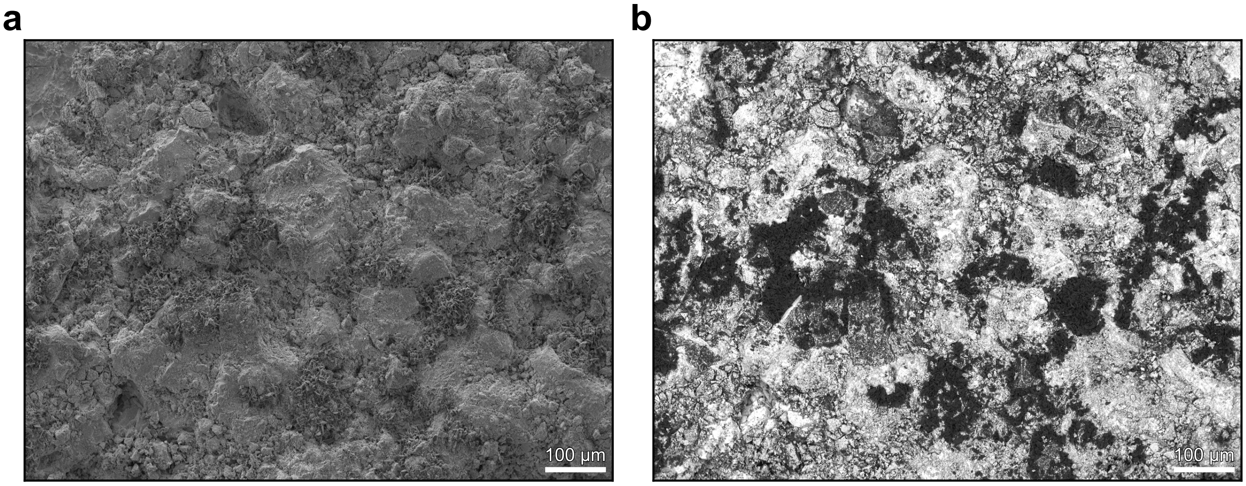

Figure S7. Bi-Bi_2K electrode SEM. (a) Secondary electron (SE) image. (b) Backscattered electron (BSE) image, revealing contrast between the two phases.

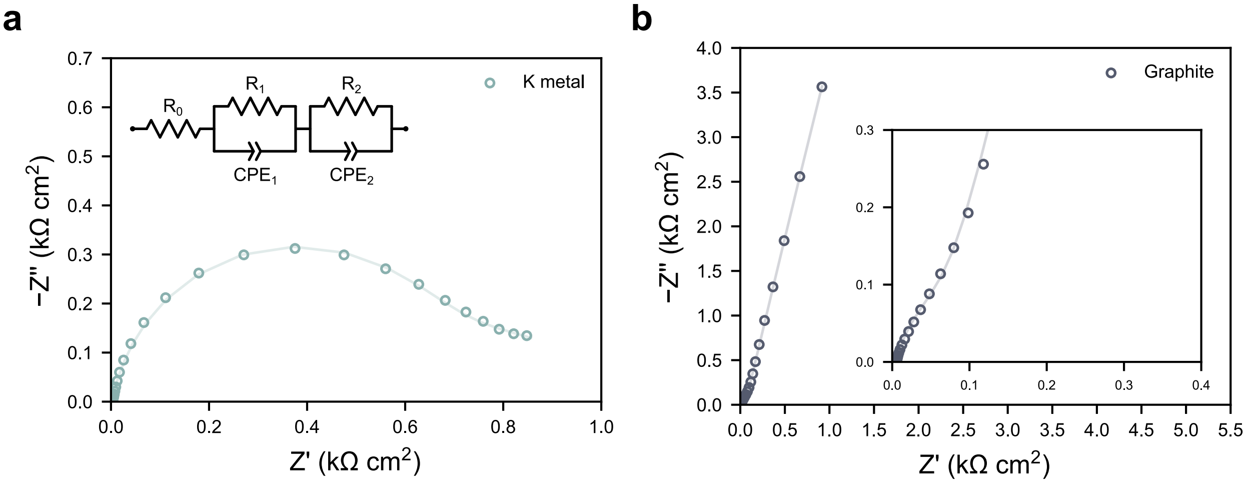

Figure S8. Three-electrode impedance fitting. (a) Potassium metal impedance (normalized by geometric area) fitted with the equivalent circuit in the inset, where $R_1 + R_2$ represents the total interfacial resistance. Fitting results are presented in Table S1. (b) Graphite impedance (normalized by geometric area) fitted with the same equivalent circuit as (a), where R_1 may result from cation migration through a surface layer and R_2 represents charge-transfer resistance. Fitting results are presented in Table S2. Fitting was performed with impedance.py.[6]

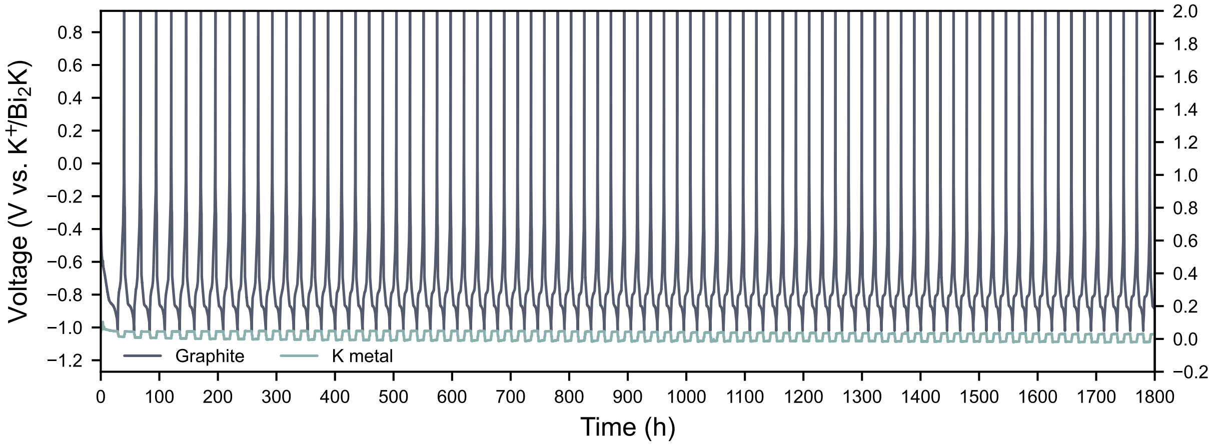

Figure S9. Extended three-electrode cycling. Voltage profiles of both the graphite and potassium metal electrodes over time during extended cycling.

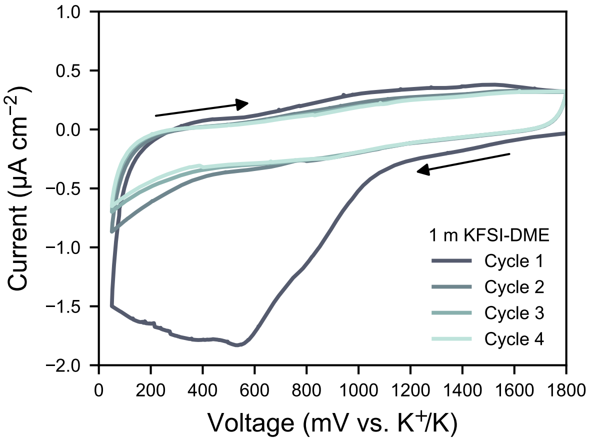

Figure S10. Cyclic voltammetry in 1 m KFSI-DME. CV curves of an aluminum electrode at 100 μV s^{-1} at 30°C.

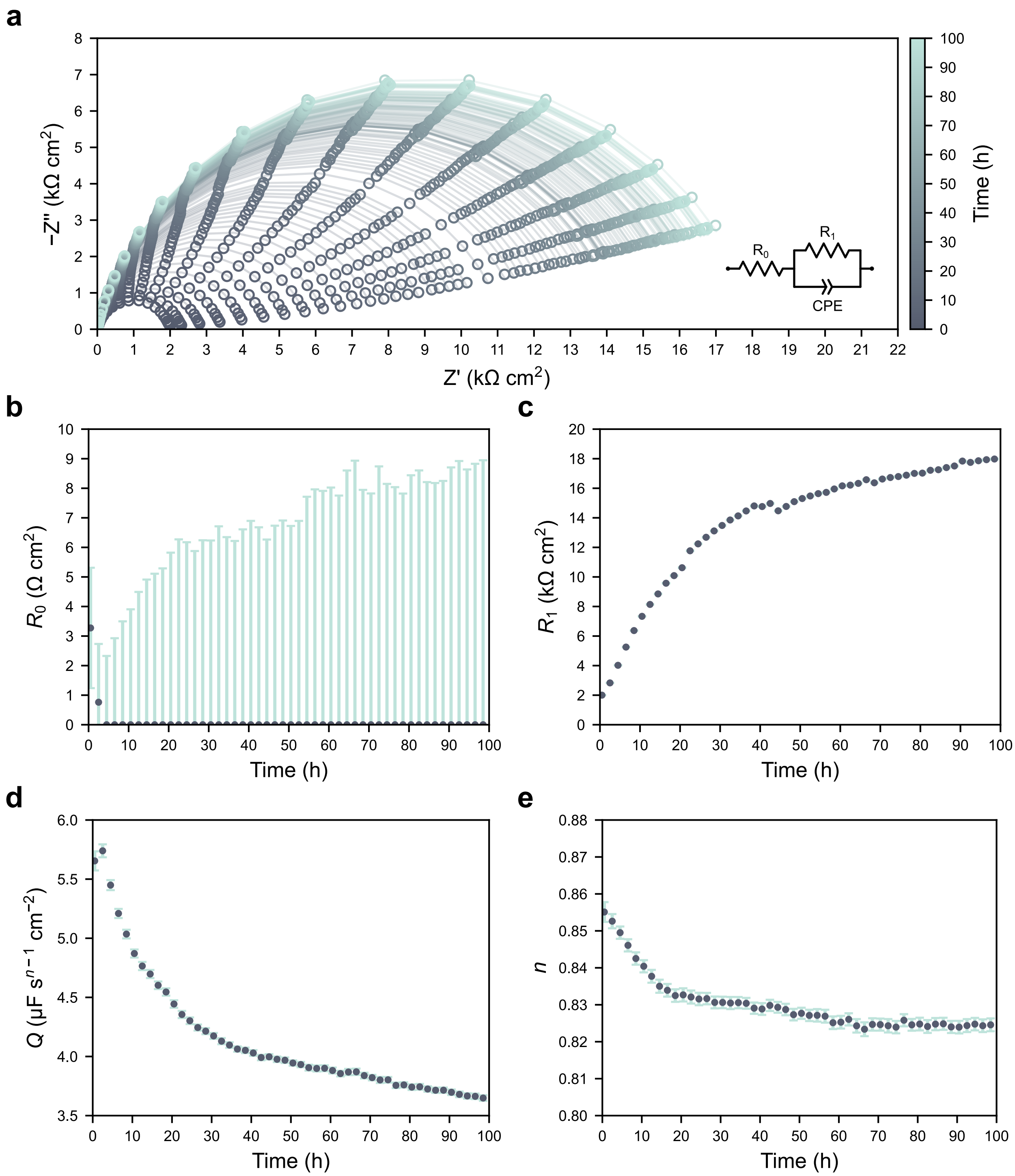

Figure S11. Exemplar K||K impedance spectra. (a) Impedance spectra measured during rest (normalized by geometric area) fitted with the equivalent circuit in the inset using impedance.py.[6] (b) Fitted series resistance, R_0 values. (c) Fitted interfacial resistance, R_1 values. (d) Fitted constant-phase element Q values. (e) Fitted constant-phase element n values. Measurements were performed in 2 m KFSI-TEP at 30°C.

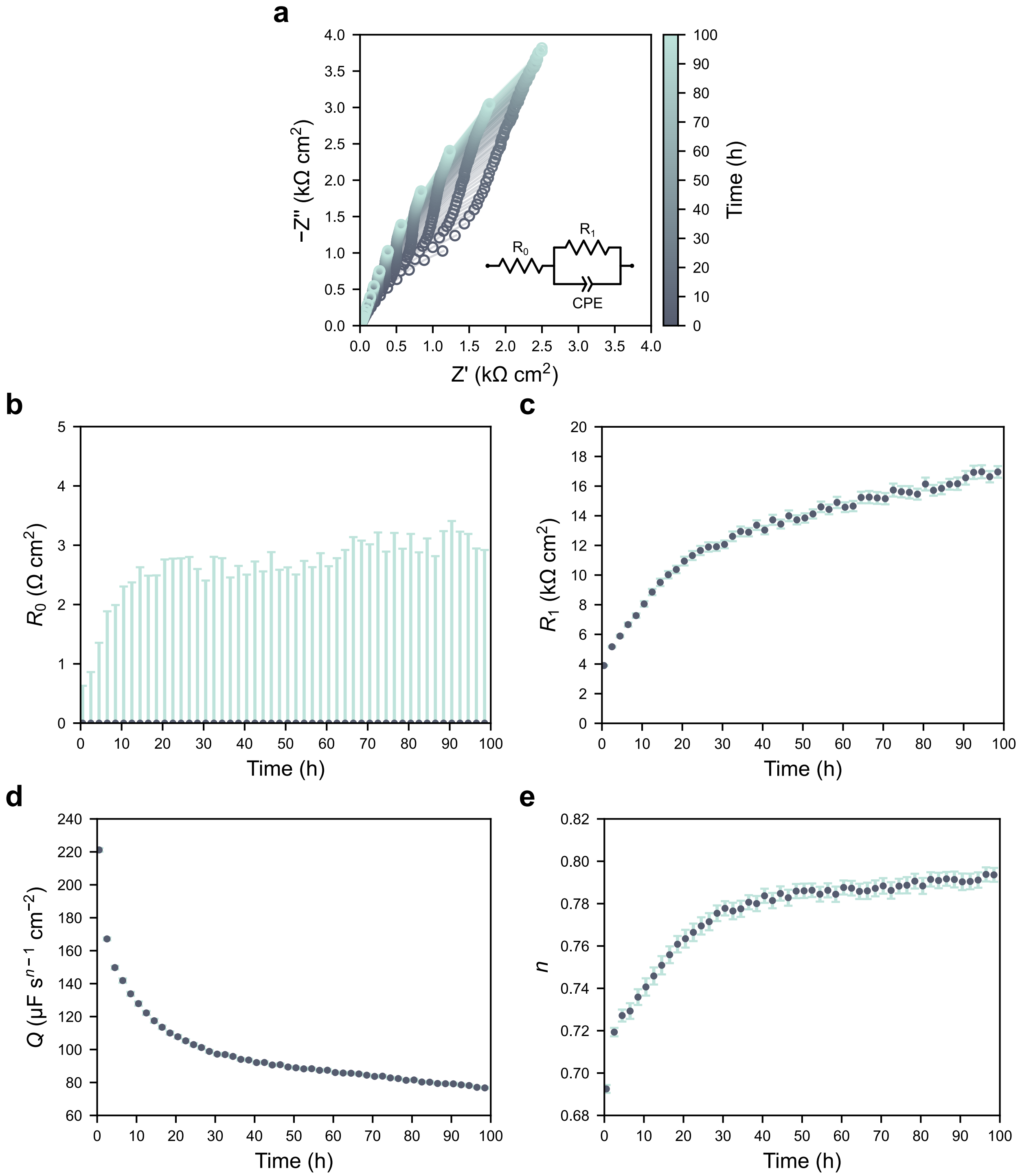

Figure S12. Exemplar Bi-Bi_2K||Bi-Bi_2K impedance spectra. (a) Impedance spectra measured during rest (normalized by approximate geometric area) fitted with the equivalent circuit in the inset using impedance.py.[6] (b) Fitted series resistance, R_0 values. (c) Fitted interfacial resistance, R_1 values. (d) Fitted constant-phase element Q values. (e) Fitted constant-phase element n values. Measurements were performed in 2 m KFSI-TEP at 30°C.

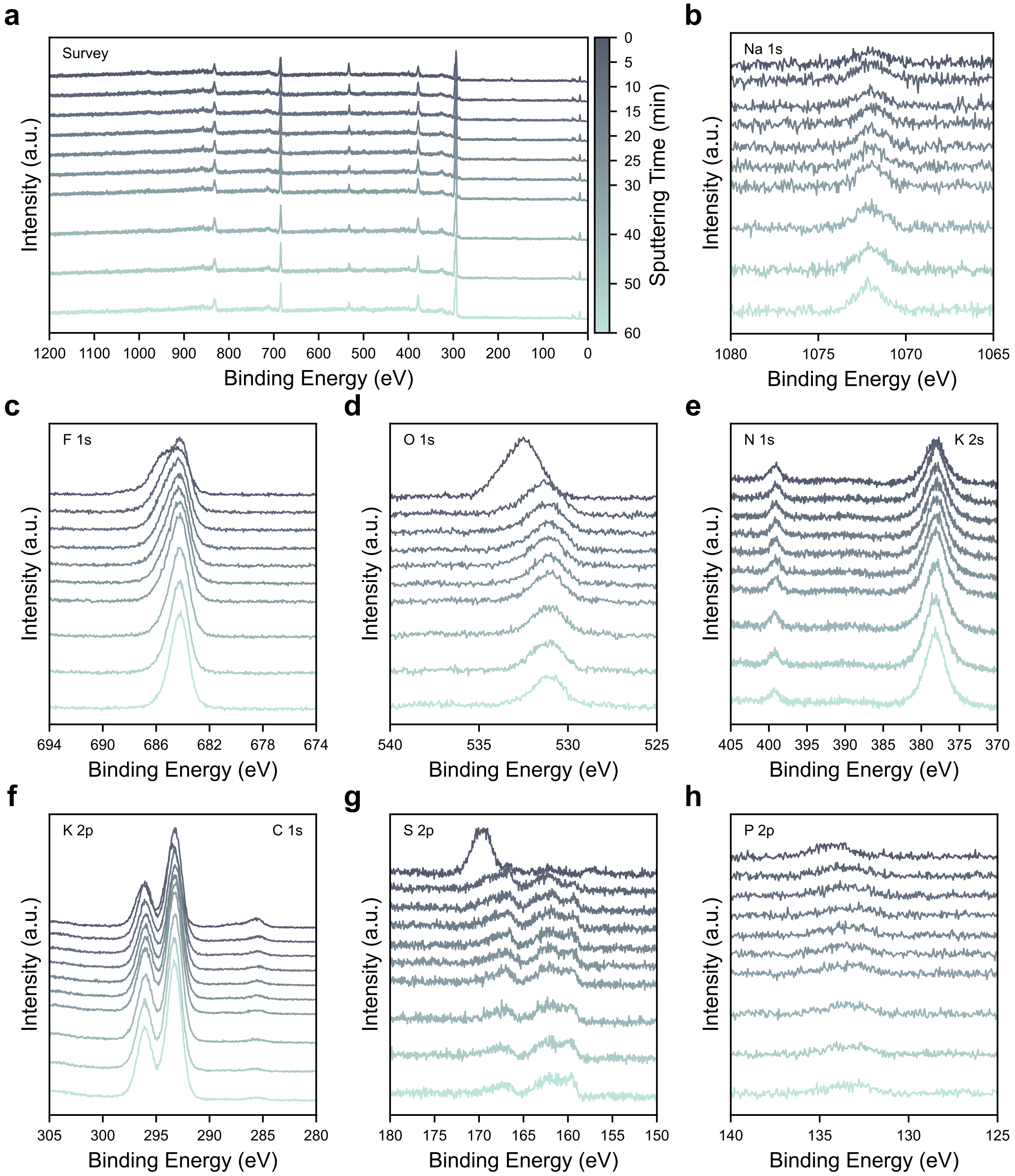

Figure S13. Full potassium metal SEI XPS spectra. (a) Survey spectra. (b) Na 1s region. (c) F 1s region. (d) O 1s region. (e) N 1s and K 2s regions. (f) K 2p and C 1s regions. (g) S 2p region. (h) P 2p region. The SEI was formed by submersion in 2 m KFSI-TEP for 20 h at 30°C.

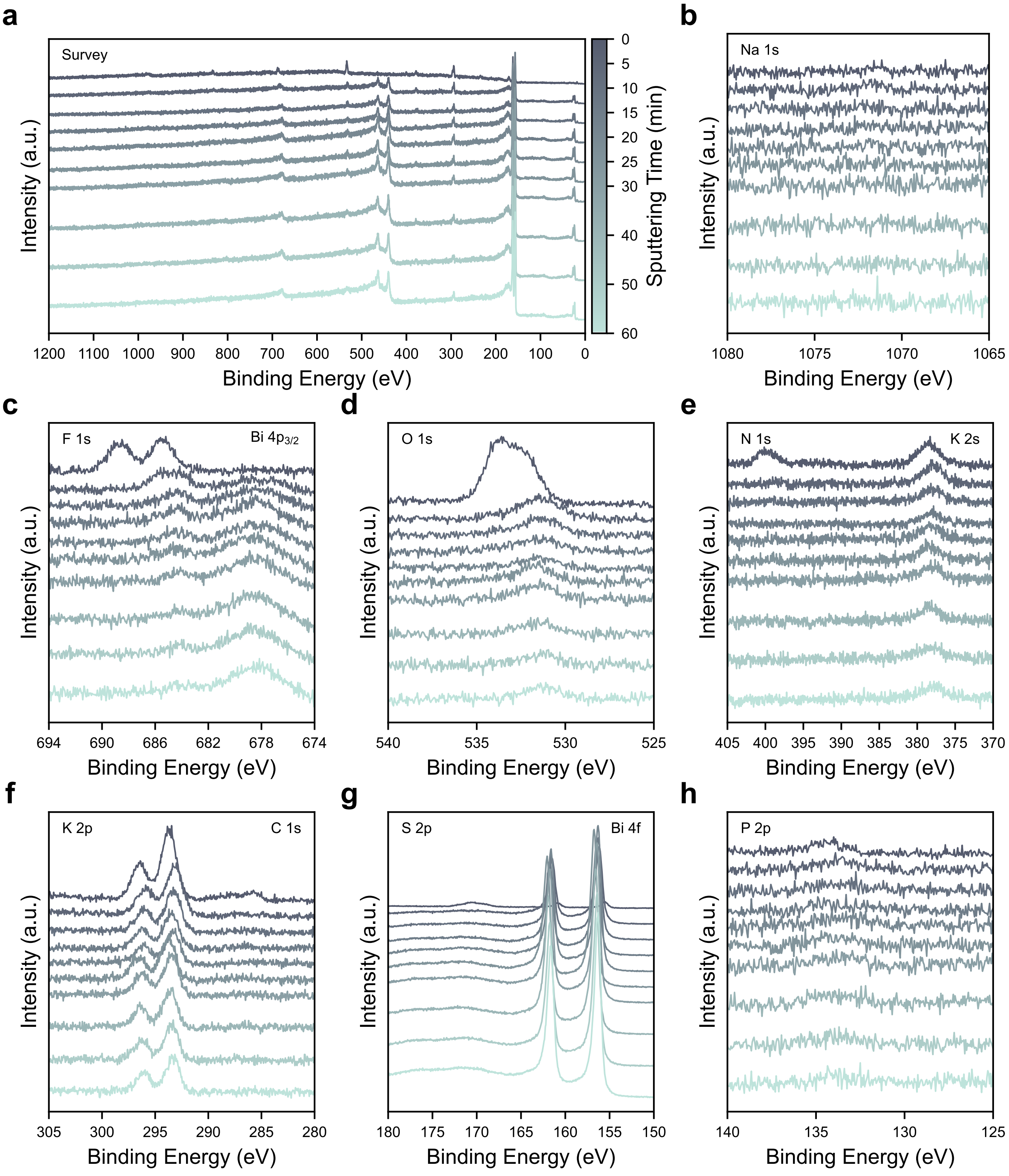

Figure S14. Full Bi-Bi_2K SEI XPS spectra. (a) Survey spectra. (b) Na 1s region. (c) F 1s and Bi $4p_{3/2}$ regions. (d) O 1s region. (e) N 1s and K 2s regions. (f) K 2p and C 1s regions. (g) S 2p and Bi 4f regions. (h) P 2p region. The SEI was formed by submersion in 2 m KFSI-TEP for 20 h at 30°C.

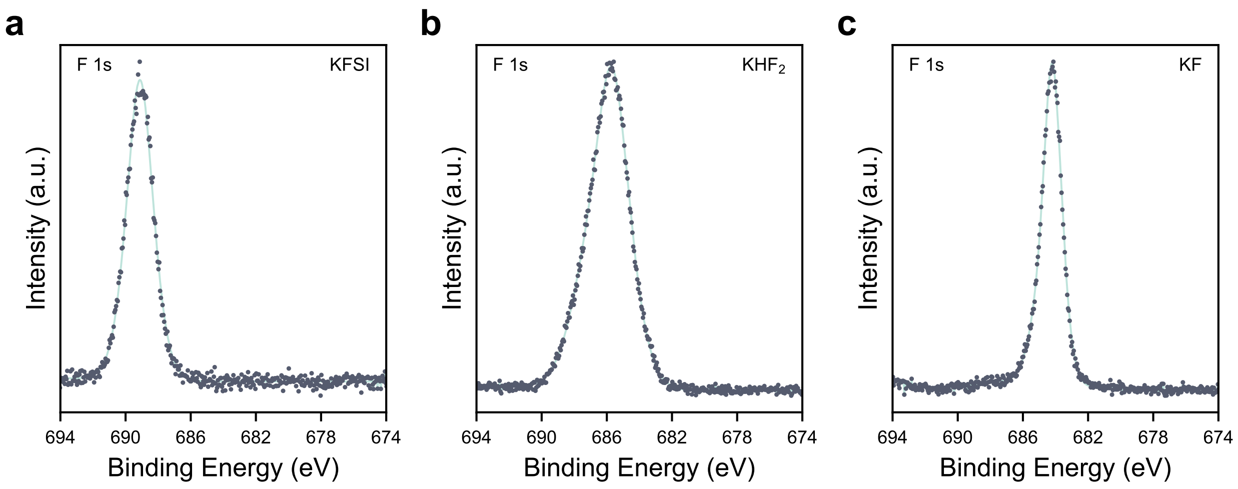

Figure S15. XPS on reference samples. (a) F 1s spectrum of KFSI. (b) F 1s spectrum of KHF_2. (c) F 1s spectrum of KF.

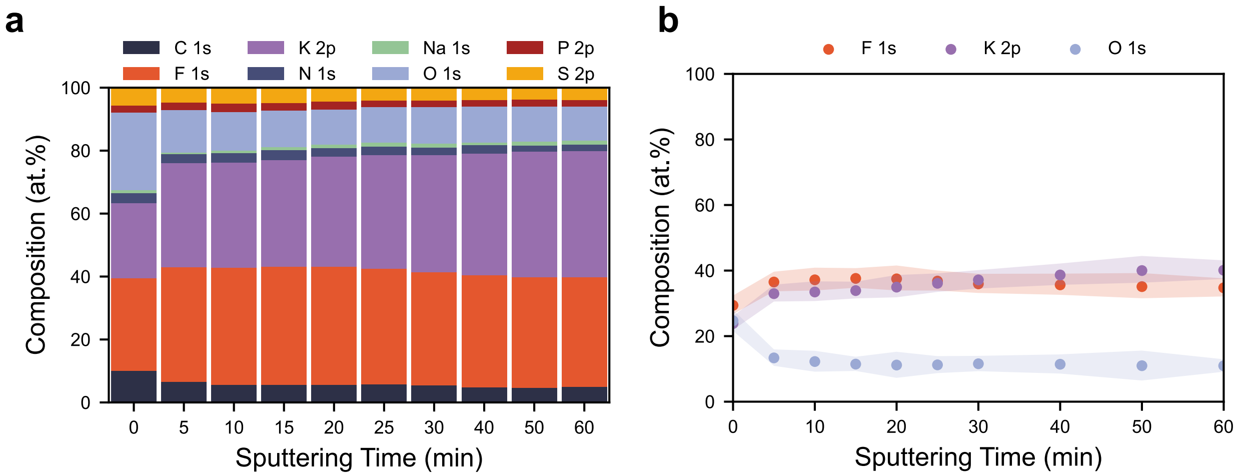

Figure S16. Potassium metal SEI quantification. (a) Composition as a function of sputtering time. (b) Concentrations of fluorine, potassium and oxygen as a function of sputtering time, with error bars taking into account the peak fitting uncertainty determined by CasaXPS.[10]

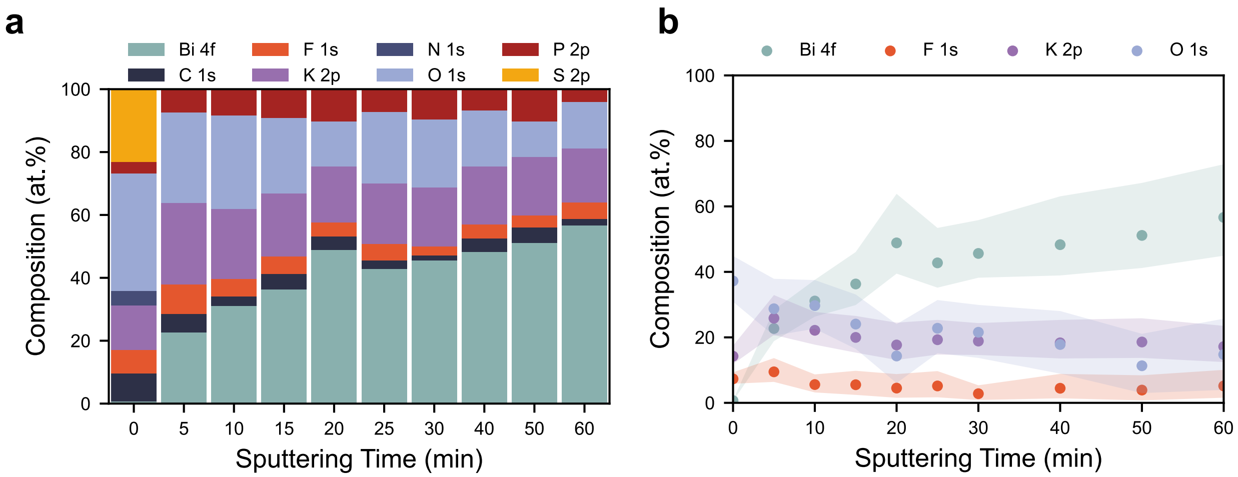

Figure S17. Bi-Bi_2K SEI quantification. (a) Composition as a function of sputtering time. (b) Concentrations of bismuth, fluorine, potassium and oxygen as a function of sputtering time, with error bars taking into account the peak fitting uncertainty determined by CasaXPS.[10]

Supporting Tables

Table S1. Potassium metal three-electrode EIS fitting. Parameters determined from the equivalent circuit model fitting of the potassium metal impedance presented in Figures 4b and S8a using impedance.py.[6]

Parameter	Value
R_0 (Ω cm^2)	1.3 ± 0.8
R_1 (Ω cm^2)	645 ± 25
Q_1 (µF s$^{n_1-1}$ cm^{-2})	20.8 ± 0.5
n_1	0.91 ± 0.01
R_2 (Ω cm^2)	405 ± 45
Q_2 (mF s$^{n_2-1}$ cm^{-2})	1.1 ± 0.1
n_2	0.62 ± 0.04

Table S2. Graphite three-electrode EIS fitting. Parameters determined from the equivalent circuit model fitting of the graphite electrode presented in Figures 4b and S8b using impedance.py.[6]

Parameter	Value
R_0 (Ω cm^2)	6 ± 0.6
R_1 (Ω cm^2)	53 ± 2
Q_1 (µF s$^{n_1-1}$ cm^{-2})	73 ± 14
n_1	0.93 ± 0.03
R_2 (kΩ cm^2)	450 ± 100
Q_2 (µF s$^{n_2-1}$ cm^{-2})	102.2 ± 0.2
n_2	0.855 ± 0.001

Supporting References

1. Dhir, S., Jagger, B., Maguire, A. & Pasta, M. Fundamental Investigations on the Ionic Transport and Thermodynamic Properties of Non-aqueous Potassium-Ion Electrolytes. *Nat. Commun.* **14,** 3833 (2023).
2. Aspinall, J., Armstrong, D. E. J. & Pasta, M. EBSD-coupled Indentation: Nanoscale Mechanics of Lithium Metal. *Mater. Today Energy* **30** (2022).
3. Coelho, A. A. TOPAS-Academic, V6. Coelho Software: Brisbane (2016).
4. Bruzzone, G. The $D1_3$ Structure Type in Intermetallic Compounds. *Acta Crystallogr. B* **25,** 1206–1207 (1969).
5. Lei, K., Wang, C., Liu, L., Luo, Y., Mu, C., Li, F. & Chen, J. A Porous Network of Bismuth Used as the Anode Material for High-Energy-Density Potassium-Ion Batteries. *Angew. Chem. Int. Ed. Engl.* **57,** 4687–4691 (2018).
6. Murbach, M. D., Gerwe, B., Dawson-Elli, N. & Tsui, L.-k. impedance.py: A Python Package for Electrochemical Impedance Analysis. *J. Open. Source Softw.* **5,** 2349 (2020).
7. Nečas, D. & Klapetek, P. Gwyddion: An Open-Source Software for SPM Data Analysis. *Open Phys.* **10,** 181–188 (2012).
8. Schneider, C. A., Rasband, W. S. & Eliceiri, K. W. NIH Image to ImageJ: 25 Years of Image Analysis. *Nat. Methods* **9,** 671–675 (2012).
9. Oliver, W. C. & Pharr, G. M. An Improved Technique for Determining Hardness and Elastic Modulus Using Load and Displacement Sensing Indentation Experiments. *J. Mater. Res.* **7,** 1564–1583 (1992).
10. Fairley, N., Fernandez, V., Richard-Plouet, M., Guillot-Deudon, C., Walton, J., Smith, E., Flahaut, D., Greiner, M., Biesinger, M., Tougaard, S., Morgan, D. & Baltrusaitis, J. Systematic and Collaborative Approach to Problem Solving Using X-ray Photoelectron Spectroscopy. *Appl. Surf. Sci. Adv.* **5,** 100112 (2021).
11. Caracciolo, L., Madec, L. & Martinez, H. XPS Analysis of K-based Reference Compounds to Allow Reliable Studies of Solid Electrolyte Interphase in K-ion Batteries. *ACS Appl. Energy Mater.* **4,** 11693–11699 (2021).
